# Mapping Differentiation under Mixed Culture Conditions Reveals a Tunable Continuum of T Cell Fates

**DOI:** 10.1371/journal.pbio.1001616

**Published:** 2013-07-30

**Authors:** Yaron E. Antebi, Shlomit Reich-Zeliger, Yuval Hart, Avi Mayo, Inbal Eizenberg, Jacob Rimer, Prabhakar Putheti, Dana Pe'er, Nir Friedman

**Affiliations:** 1 Department of Immunology, Weizmann Institute of Science, Rehovot, Israel; 2 Department of Molecular Cell Biology, Weizmann Institute of Science, Rehovot, Israel; 3 Transplantation Institute and Department of Medicine, Beth Israel Deaconess Medical Center, Harvard Medical School, Boston, Massachusetts, United States of America; 4 Department of Biological Sciences, Columbia University, New York, New York, United States of America; University of Pennsylvania, United States of America

## Abstract

Cell differentiation is typically directed by external signals that drive opposing regulatory pathways. Studying differentiation under polarizing conditions, with only one input signal provided, is limited in its ability to resolve the logic of interactions between opposing pathways. Dissection of this logic can be facilitated by mapping the system's response to mixtures of input signals, which are expected to occur *in vivo*, where cells are simultaneously exposed to various signals with potentially opposing effects. Here, we systematically map the response of naïve T cells to mixtures of signals driving differentiation into the Th1 and Th2 lineages. We characterize cell state at the single cell level by measuring levels of the two lineage-specific transcription factors (T-bet and GATA3) and two lineage characteristic cytokines (IFN-γ and IL-4) that are driven by these transcription regulators. We find a continuum of mixed phenotypes in which individual cells co-express the two lineage-specific master regulators at levels that gradually depend on levels of the two input signals. Using mathematical modeling we show that such tunable mixed phenotype arises if autoregulatory positive feedback loops in the gene network regulating this process are gradual and dominant over cross-pathway inhibition. We also find that expression of the lineage-specific cytokines follows two independent stochastic processes that are biased by expression levels of the master regulators. Thus, cytokine expression is highly heterogeneous under mixed conditions, with subpopulations of cells expressing only IFN-γ, only IL-4, both cytokines, or neither. The fraction of cells in each of these subpopulations changes gradually with input conditions, reproducing the continuous internal state at the cell population level. These results suggest a differentiation scheme in which cells reflect uncertainty through a continuously tuneable mixed phenotype combined with a biased stochastic decision rather than a binary phenotype with a deterministic decision.

## Abstract 2

An experimental and theoretical study of T cell differentiation in response to mixed-input conditions reveals that cells can tune between Th1 and Th2 states through a continuum of mixed phenotypes.

## Author Summary

During cell differentiation, progenitor cells respond to external signals that drive the expression of genes that are characteristic of the differentiated cell states. This process is controlled by gene regulatory networks that typically involve positive autoregulation and cross-inhibition between master regulators of the two differentiated states. Mapping the system's response to mixtures of external signals can help us to understand the operational logic of these binary cell fate decisions. Here, we study differentiation of CD4^+^ T cells into Th1 and Th2 lineages under mixed-input conditions, at the single cell level. We reveal that cell state is not restricted to a small number of well-defined phenotypes, but rather tunes through a continuum of mixed-phenotype states in which levels of lineage-specifying transcription factors gradually change with the levels of the two inputs. Using mathematical modeling we establish the conditions under which the system has one stable steady state that continuously tunes in response to changes in levels of the inputs. Results of this model qualitatively explain our experimental observations. We further characterize expression patterns of downstream lineage-specific genes—cytokines that are driven by the two master regulators upon cell re-stimulation. We find a highly heterogeneous population with cells expressing either one of the cytokines, both cytokines, or neither. Of note, the fraction of cells in these subpopulations continuously tunes with input levels, thus reproducing a tunable state at the cell population level. Our results can be explained by a two-stage scheme in which the gene regulatory network is responsible for a continuously tunable cell state, which is translated into a heterogeneous cytokine expression pattern through uncorrelated and biased stochastic processes.

## Introduction

**Figure 1:**
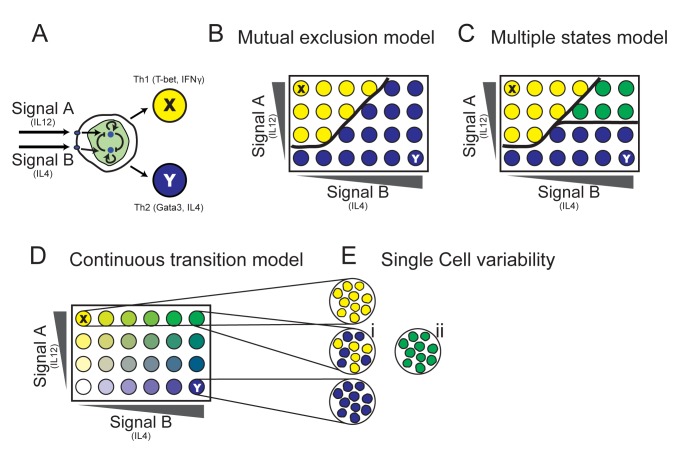
Understanding the logic of cell fate decisions by studying response to a matrix of input combinations at the single cell level. (A) A schematic representation of cell differentiation through a binary cell fate decision. Signal A drives differentiation of a precursor cell into the differentiated state X. Signal B drives it into the state Y. Cell decision is mediated by a GRN that typically involves interacting signaling pathways that contain various positive and negative feedback loops. (B) Mutual exclusion model: The GRN has two stable states, each corresponding to the phenotype of a specific differentiated lineage. (C) Multiple states model: In a range of input conditions cells are found in a third state, co-expressing characteristic genes of both lineages. (D) Continuous transition model: As the input conditions vary, a single steady state continuously shifts between the two extreme phenotypes, giving rise to a continuum of differentiated cell states with mixed characteristics. (E) Single cell variability: Under mixed input conditions, the cell population can be either heterogeneous, with each cell in either the X or Y “pure state” (i), or in a “mixed state,” with cells co-expressing lineage-specific factors (ii). Note that under polarizing input conditions (top-left and bottom-right corners), all models are indistinguishable.

Consider a general cell differentiation process in which precursor cells can respond to two external signals, each driving differentiation into a specific lineage (Figure 1A). Such processes are common, for example, in stem-cell differentiation in the early embryo [1],[2] and in the hematopoietic system in which more specialized cells are generated from earlier progenitors through cascades of binary cell fate decisions [3]. Under mixed conditions, when both driving signals are present, several hypothetical outcomes may occur. If the two differentiated states are mutually exclusive, cells will make a definite decision and will differentiate into one state or the other (Figure 1B). Most experimental and theoretical studies of cell differentiation show occurrence of such mutually exclusive steady states [4]–[6]. Another scenario shown by other models is that of multistability (Figure 1C), where some input conditions give rise to a third steady state in which genes specific to both lineages are co-expressed. Tri-stability was observed in a number of systems in which low-level co-expression of lineage-specific transcription factors occurs in progenitor cells [7]–[9]. In both scenarios the transition between states is sharp. In contrast, cell state can also shift continuously from one extreme to the other (Figure 1D). Such gradual transition at the population level can be realized in qualitatively different ways at the single cell level. Each cell on its own can make a definite decision, resulting in a heterogeneous population with cells showing either one or the other phenotype (Figure 1Ei). Alternatively, cells can show a mixed phenotype at the single cell level, with individual cells co-expressing specific genes of both lineages simultaneously (Figure 1Eii). Notably, all scenarios presented in Figure 1 are indistinguishable under polarized input conditions—that is, when applying only one input at a time.

The understanding of binary cell fate decisions in various cellular systems has advanced in the last decade through combination of experimental investigations with mathematical modelling [3],[5],[6],[9]. Dynamical systems theory was used for describing how gene regulatory networks (GRNs) that control cell differentiation influence cell state over time. This type of analysis provides a framework for defining differentiated states as attractors (stable steady states) of a dynamical system that describes the GRN [3],[10]. A simplified GRN motif was identified in most studied binary cell fate systems, in which two fate determining transcription factors (corresponding for the two differentiated lineages) cross-inhibit each other, while each factor also positively regulates its own level (Figure 1A). This motif was investigated, for example, in the PU.1-GATA1 system controlling erythroid/myeloid cell differentiation [9],[11]–[14], and also in the Th1–Th2 system [5],[6],[15]–[17], which is the subject of the current study. It was shown by these studies and others that this network motif, under various conditions, can give rise to either a bi-stable or a tri-stable system. In the latter case, two steady states correspond to the differentiated lineages, and another steady state corresponds to the progenitor cell state, in which both TFs are expressed at intermediate levels.

While cell differentiation was traditionally considered as a binary process, recent studies of hematopoietic cells reveal existence of a continuum of cell states bridging previously described subsets [18],[19]. However, it is still not well understood how such intermediate cell states are generated from progenitor states, nor how their existence complies with prevailing theoretical models of cell differentiation. In order to gain better understanding of the logic employed by the GRN governing cell differentiation, it is beneficial to study its responses under mixed input conditions [2],[20]–[25] at the single cell level.

In this study we use differentiation of naive CD4^+^ T cells towards the Th1 and Th2 lineages as a model system to study this question. Antigen-activated CD4^+^ T cells can differentiate into various cell types depending mainly on the cytokines present in their environment during activation [26],[27]. Differentiation of CD4^+^ T cells towards the Th1 lineage is driven by the cytokine IL-12, while IL-4 drives differentiation towards the Th2 lineage (Figure 1A). Th1 cells, involved in protection against intracellular pathogens, are characterized by the expression of the lineage-specific transcription factor (TF) T-bet, and by production and secretion of effector cytokines such as IFN-γ and TNFα [26]. Th2 cells express the lineage-specific TF, GATA3; secrete the cytokines IL-4, IL-5, and IL-13; and are involved in protection against extracellular pathogens [26]. Existence of cells co-expressing IFN-γ and IL-4 was observed in both mouse and human [28],[29], but the input conditions and the status of expression of transcription factors leading to their formation are not clear.

## Results

### Mapping Expression of T-Bet and GATA3 under Mixed Input Conditions Reveals a Continuously Tuneable Mixed State

In order to characterize the differentiation decision logic, naïve CD4^+^ T cells were activated in the presence of a combinatorial matrix of the two external signals IL-12 and IL-4: increasing levels of IL-12 (signal A) in rows and of IL-4 (signal B) in columns (see also Text S1). Following 7 d of culture, cells were restimulated through their T cell receptor, and their responses were measured. We characterize each cell by four parameters: the levels of the two lineage-specific TFs, T-bet and GATA3, and the levels of the two lineage characteristic cytokines, IFN-γ and IL-4. Levels of these four proteins were measured for each cell by intracellular staining using fluorescently labelled monoclonal antibodies, followed by flow cytometry.

**Figure 2:**
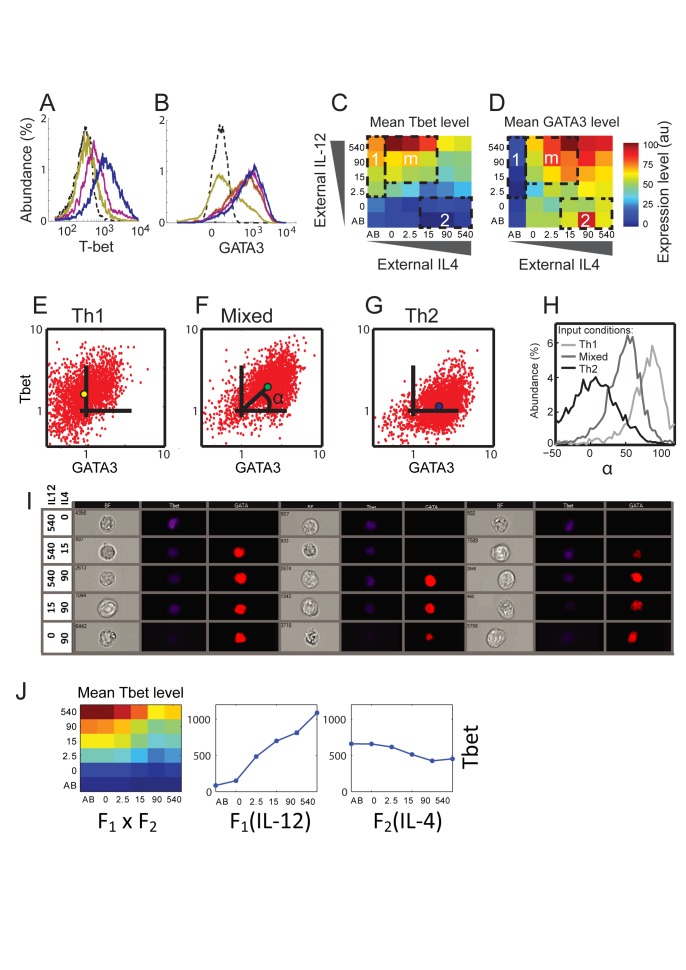
Input function for Th1/Th2 cell differentiation under mixed input conditions reveals a tunable mixed phenotype. (A) Histograms of T-bet levels measured in populations of cells cultured with decreasing levels of IL-4 and increasing levels of IL-12 (yellow to blue color). Dashed curve, cells from a T-bet knockout mouse. (B) Histograms of GATA3 levels measured in populations of cells cultured with increasing levels of IL-4 and a constant level of IL-12 (yellow to blue color). Dashed curve, GATA3 isotype control staining. (C, D) Measured median fluorescence intensities (MFI) for T-bet (C) and GATA3 (D), in response to a matrix of orthogonal gradients of the two input signals IL-12 and IL-4. Regions 1 and 2 represent standard polarizing conditions used to generate a Th1 or Th2 response, respectively. Region m represents a state with mixed inputs, resulting in expression of both T-bet and GATA3. (E–G) Scatter plots showing normalized measured expression patterns of T-bet and GATA3, under the conditions marked by 1,m,2 in panels (C, D) (see Text S1 for details). A single, unimodal population is observed, which shifts in the T-bet-GATA3 plane in response to input signals. Colored dots in each panel show the population median. (H) Distributions of the parameter α, representing the ratio between expression levels of T-bet and GATA3 (see F and definition in the main text), for cell populations cultured under Th1, Th2, and mixed input conditions. The distributions all show a single peak, and continuously shift from a Th1 (α≈90°) to a Th2 state (α≈0°). (I) Representative images of cells cultured under various input conditions as indicated, fixed and stained for T-bet (blue, pseudo-color) and GATA3 (red, pseudo-color). Images were acquired using fluorescent flow microscopy (see Materials and Methods). Three cells for each condition are shown in the bright-field (BF), T-bet, and GATA3 channels. (J) The input function of T-bet is well described by separation of variables, with each input influencing the output in an independent manner. Shown are the calculated dependencies of T-bet on the two inputs, F_1_(IL-12) and F_2_(IL-4), and the calculated input function given by F_1_(IL-12)×F_2_(IL-4), which shows a high similarity with the measured data (C). See Figure S3 for similar results for GATA3, IFN-γ, and IL-4.

First, we mapped the population average response of the TFs to a matrix of input conditions. Histograms of the levels of T-bet (Figure 2A) and GATA3 (Figure 2B) show unimodal distributions that change continuously with input signals. As expected, we measure high levels of T-bet and low levels of GATA3 in a region of inputs that corresponds to Th1 driving conditions (Figure 2C,D, region 1), and the opposite pattern for a region of Th2 driving conditions (Figure 2C,D, region 2). However, we find that expression of T-bet and of GATA3 are not mutually exclusive: both TFs are co-expressed at a relatively high level in response to a large variety of mixed input conditions (Figure 2C,D, region m). These results are supported by measurements of mRNA levels, which also show co-expression of T-bet and GATA3 at high levels under mixed input conditions (Figure S1B). Co-expression of T-bet and GATA3 arises at early time points of the differentiation process under mixed input conditions and can be observed already at day 3 after activation (Figure S2). Our results show that mouse CD4^+^ T cells can be driven into a mixed Th1–Th2 state directly from the naïve state, in addition to reprograming of Th2 cells as was recently shown [30]. Moreover, we reveal that this mixed state is continuously tuneable, showing varying levels of the lineage-specific TFs in response to different mixtures of driving signals.

Next, we characterized the response to mixed inputs at the single cell level. In Figure 2E–G, we present scatter plots showing T-bet and GATA3 levels of cells cultured under Th1 (E), mixed (F), or Th2 (G) conditions. In all cases cells cluster as a single unimodal population, and no evidence of bi-stability is observed. Importantly, under mixed conditions most cells co-express T-bet and GATA3. To better visualize single-cell patterns of expression, we define a parameter α for each cell, which is related to the ratio between its T-bet and GATA3 expression levels: *α* = atan(T-bet/GATA3)—that is the angle that the cell forms with the *x*-axis (Figure 2F, see Materials and Methods). α is a robust measure of the ratio between T-bet and GATA3 that is not prone to noise in low-value denominators. Using this ratiometric parameter reduces effects of extrinsic factors such as cell size, which influence levels of both proteins in a similar way. Plotting the distribution of this parameter (Figure 2H) reveals that external signals continuously shift the cell population from a GATA3 dominant state (α = 0°) to a T-bet dominant state (α = 90°) while exhibiting intermediate values for input mixtures. This is indicative of a population of cells that co-express the two master regulators at levels that continuously tune with inputs, though with a relatively large cell-to-cell heterogeneity. These results are supported by analysis using flow microscopy, which shows co-expression of T-bet and GATA3 at varying levels in nuclei of T cells driven under mixed input conditions, spanning the range between Th1 and Th2 states (Figure 2I).

Now that we have demonstrated a unimodal continuous output behaviour, we inquired how both inputs combine to determine this output. We find that each input (IL-12, IL-4) influences the outputs in an independent manner. The expression level of the two TFs can be described as: F_1_(IL-12)×F_2_(IL-4), where the functions F_1_ and F_2_ represent the dependence on each input separately (Figures 2J, S3). A similar property was previously observed for input functions describing bacterial promoters [31]. The resulting one-dimensional dependencies (F_1_, F_2_) are gradual, consistent with a continuous tuneable state. This separation of variables simplifies description of the system's response under mixed conditions and restricts possible mathematical models of the system. We note that levels of T-bet increase with increasing level of IL-12 and slightly decrease with increasing levels of IL-4, as can be expected. On the other hand, we find that levels of GATA3 increase with IL-4 but also slightly increase with levels of IL-12 when IL-4 is present (Figures 2D and S3). This finding indicates that IL-12 does not strongly repress GATA3, and may even have some net indirect weak positive effect on its level of expression.

### A Mathematical Model for Binary Cell Fate Decisions Complies with a Continuously Tuneable State if Feedbacks Are Gradual, Not Steep

**Figure 3:**
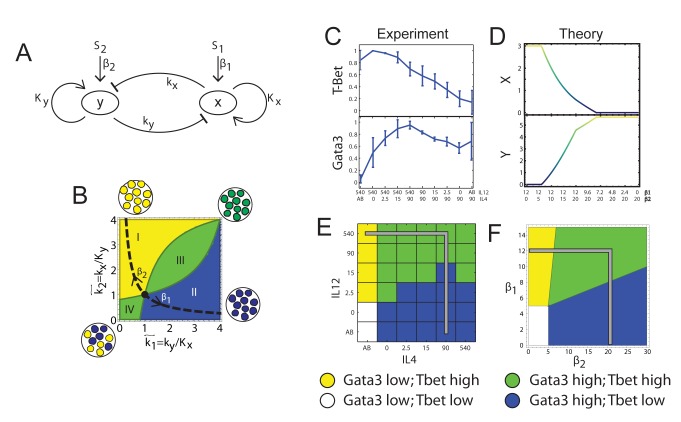
A model for a continuously tunable mixed-state under mixed input conditions. (A) A schematic model for the effective GRN module regulating Th1–Th2 differentiation. (B) Analysis of the model for gradual feedback links (*n* = 1). The number and location of fixed points for given input signals depend on the ratio between the strength of negative and positive feedbacks, 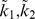 (see Text S1 for details). In region I, the GRN has a single fixed point with a high level of x and a low level of y. In region II it has a single fixed point with low x and high y. In region III it has a single fixed point with co-expression of both TFs, whereas in region IV it has two stable fixed points (bifurcation). (C–D) TF levels shift continuously upon gradual changes in input signal mixtures. Measured levels (MFI) of T-bet and GATA3 (C) along a trajectory in input plane, which interpolates between a Th1 and a Th2 condition (shown in E, gray line). Continuous changes in TF levels are in agreement with model predictions for *n* = 1, region III (D) and do not show any bi-stability or sharp transitions as predicted by a high-*n* model, or low-*n* model region IV. (E–F) Mapping patterns of TF co-expression over the entire input plane, comparing experiment (E) and model (F). For each TF, we define a threshold level T at ∼50% of its maximal expression level. Regions' color represents patterns of co-expression, as shown in the legend.

To gain a theoretical understanding of our findings of continuously tuneable cell states, we analyzed a functional network motif that is widely used for describing systems of binary cell fate decisions (Figure 3A). The motif consists of two lineage-specifying transcription factors, X and Y (corresponding to T-bet and GATA3 in the case of Th1–Th2 differentiation), which cross-inhibit each other and positively autoregulate their own expression. This network motif has been used to model Th1–Th2 differentiation [5],[6],[15],[17], as well as other cell differentiation systems [9],[11]–[14],[32]. In the case of the Th1–Th2 system, it represents a simplified view of more complex biological interactions (Figure S13), offering a tractable system that can provide general principles, rather than quantitatively explaining fine details of the data, which may require a refined model. Each link in the simplified network effectively describes more than one regulatory link of the full network. For example, T-bet autoregulation is mediated by at least two parallel pathways: IFN-γ secretion, which is up-regulated by T-bet, and in turn drives T-bet expression via STAT1 signalling; and up-regulation of IL12R by T-bet, which drives IFN-γ expression via STAT4 (Figure S13 and Table S1 for related references). GATA3 autoregulation involves both a direct regulatory effect, as well as an autocrine/paracrine loop through GATA3-mediated secretion of IL-4, which drives GATA3 expression via STAT6 (Figure S13 and Table S1). Of note, the full network of Figure S13 is coherent with respect to the links of the simplified GRN; each path in the full network that starts and ends at the T-bet node, without going through GATA3, has a positive sign; and similarly for all paths that start and end at GATA3 without going through T-bet. Each path that starts at T-bet and ends at GATA3 has a net negative sign; and similarly for all paths that start at GATA3 and end at T-bet (as detailed in Figure S13).

Previous analyses [5],[6],[9],[15],[33] showed that the motif of Figure 3A induces bi- or tri-stability (Figure 1B,C) through a toggle switch mechanism. In these studies, the regulatory links in the GRN are usually described by a steep function, e.g. a Hill function, *x^n^*/(*x^n^*+*k^n^*), with a Hill parameter *n>1*. We will now show how the same motif, with different parameterization, can recapitulate our findings of a continuous transition with one stable state.

Based on our observations of graded responses, we analysed the steady states of the GRN of Figure 3A under conditions of gradual regulatory links, using a Hill parameter of *n* = 1. We find that for given levels of the two inputs, β1 and β2, the system's behaviour depends on the ratios between the threshold levels of the cross-inhibitory and the autoregulatory arrows (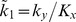, 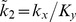, respectively; see Figure 3A,B, Figure S12, and Text S1). If these parameters correspond to the area below the dashed hyperbola in Figure 3B (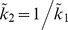), the system is either mono-stable at one of the extreme phenotypes (one TF highly expressed and the other at zero, Figure 3B regions I and II), or bi-stable (region IV). However, if the parameters correspond to the area above the hyperbola, the system always has only one stable state. This state continuously tunes between the extreme phenotypes through region III, by changing the ratio of the two inputs, β1/β2. Inside region III, both TFs are expressed at intermediate levels. Changing inputs without changing internal parameter values for the GRN cannot shift a bi-stable system into a mono-stable one and vice versa.

Our experimental observations support the low-*n* model in a number of ways. First, we plot T-bet and GATA3 levels for a trajectory in input-space that corresponds to gradually changing the ratio β1/β2. Expression levels of both TFs continuously shift from a pure Th1 state into a pure Th2 state, without sharp transitions (Figure 3C), in accordance with model predictions (Figure 3D). Moreover, experimental results concur with the model over the entire measured input-space (Figure 3E,F). Finally, multistability is expected to result in either a multimodal population at transition points, or increased levels of noise in intermediate expression levels [34],[35]. Analysis of expression-level distributions of T-bet and GATA3 does not support bi-modality of the population (Figures 2A and S1A). Additionally, the noise level, calculated as SD/mean, does not considerably change with varying input conditions, for both T-bet and GATA3 (Figure S4). We thus conclude that the accepted core model for the GRN controlling cell differentiation can comply with our observations for a mixed and mono-stable tuneable state under mixed conditions, provided that the effective regulatory links gradually depend on the levels of the regulatory proteins. In particular, a low hill parameter of the autoregulatory links is sufficient, under most parameter values, to account for this behaviour (see Text S1), while cross-inhibition can be steep. Additionally, we predict that the effective positive autoregulatory links in the network motif of Figure 3A are dominant over cross-inhibition so that the system resides “above the hyperbola” of Figure 3B.

### Expression of Lineage-Specific Cytokines: A Highly Heterogeneous Cell Population with a Continuously Tuneable Mean Behaviour

**Figure 4:**
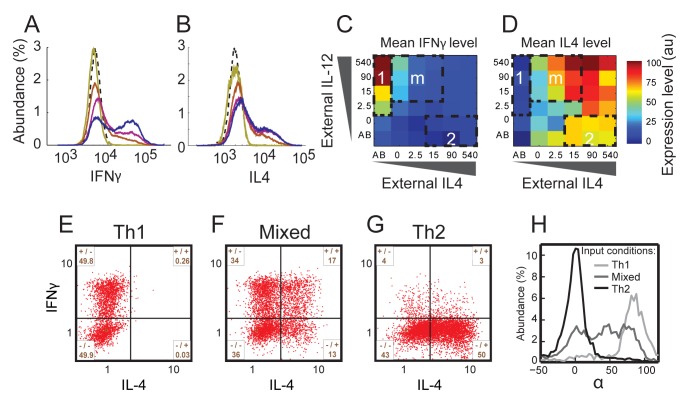
Mapping input function of cytokine expression reveals a highly heterogeneous population under mixed input conditions. (A) Histograms of IFN-γ secretion levels measured in a population of cells cultured with decreasing levels of IL-4 and increasing levels of IL-12 (bright to dark colour). Dashed curve, isotype control. (B) Histograms of IL-4 secretion levels measured in a population of cells cultured with increasing levels of IL-4 and a constant level of IL-12 (yellow to blue colour). Dashed curve, isotype control. (C, D) Measured MFI for IFN-γ (C) and IL-4 (D), in response to a matrix of orthogonal gradients of the two input signals IL-12 and IL-4. Regions 1, 2, and m are the same as in Figure 2C,D. (E–G) Scatter plots showing normalized measured expression patterns of IFN-γ and IL-4. Under mixed conditions cell population is highly heterogeneous in cytokine expression, with subpopulations expressing only IFN-γ (+/−), only IL-4 (−/+), both cytokines (+/+), and neither one (−/−). (H) Distributions of the parameter α′, representing the ratio between expression levels of IFN-γ and IL-4 (see definition in the main text) for cell populations cultured under Th1, Th2, and mixed input conditions. As for the TFs, the distributions under Th1 and Th2 conditions show a single peak. However, in contrast with TF, under mixed input conditions the distribution is broad and covers the whole range of values between α′ = 90° (Th2) and α′ = 0° (Th1).

We further characterized cells' phenotype by mapping the levels of the two lineage characteristic cytokines IFN-γ and IL-4 over the entire input space, asking to what extent do they follow our findings regarding the TFs. In contrast with the TFs, the expression-level distributions of these cytokines are bimodal (Figure 4A,B), which is a well-known characteristic of cytokine gene expression [36]. The fraction of cytokine-expressing (positive) cells varies with input level, while the level of cytokine expression for these positive cells remains almost constant (Figure 4A,B). Despite this difference, the population mean follows a pattern similar to that of the TFs over the different input mixtures as observed both by internal staining (Figure 4C,D and Figure S2, Pearson correlation 0.56 (0.91) between IFN-γ and T-bet (IL-4 and GATA3), respectively) and ELISA (Figures S2 and S5, Pearson correlation 0.75 (0.65)). A mixed phenotype is observed also here, as co-expression of IFN-γ and IL-4 is evident under mixed conditions at the protein (Figure 4C,D) and mRNA (Figure S1D) levels.

As with the master regulators, cytokine input functions can also be described as separable functions of the two inputs (Figure S3). Notably, IFN-γ protein levels show a sharper negative response to external IL-4 compared with that of T-bet. This might reflect the more direct repression of IFN-γ by GATA3, which then indirectly down-regulates T-bet [37].

Single-cell analysis (Figure 4E–G) reveals a highly heterogeneous expression of IFN-γ and IL-4 under mixed input conditions, with subpopulations of cells expressing only IFN-γ, only IL-4, both cytokines or neither, as shown in Figure 4F. Consistent with the tuneable state observed at the TF level, input signals also continuously modulate the percentage of cells in each subpopulation of cytokine co-expression (Figure S6). Similar to the analysis above, we define for each cell a parameter α′, which is related to the ratio between its IFN-γ and IL-4 expression levels: α′ = atan(IFN-γ/IL-4). Under Th1 and Th2 driving conditions, α′ is peaked around 90° and 0°, respectively, as expected. However, under mixed input conditions α′ shows a very broad distribution, significantly overlapping with both Th1 and Th2 populations, reflecting the large heterogeneity in levels of cytokine expression (Figure 4H).

To investigate the behaviour of other lineage-specific cytokines, we repeated these experiments measuring also levels of the Th2 cytokines IL-5 and IL-13 (total of six parameters for each cell). Under our experimental conditions we observed only a small fraction of cells expressing IL-5 (∼10% under Th2 conditions versus ∼1% under Th1 conditions), which didn't allow us to significantly analyze its co-expression patterns. IL-13 showed a very similar behaviour to that of IL-4. Under mixed conditions there is a subpopulation of cells co-expressing IL-13 and IFN-γ (Figure S17), and the mean level of both cytokines continuously increases by shifting input conditions from Th1 to Th2 through various mixtures (Figure S18).

### Cytokine Expression Can Be Described as Independent Stochastic Processes That Are Biased by Input Conditions and Transcription Factor Levels

**Figure 5:**
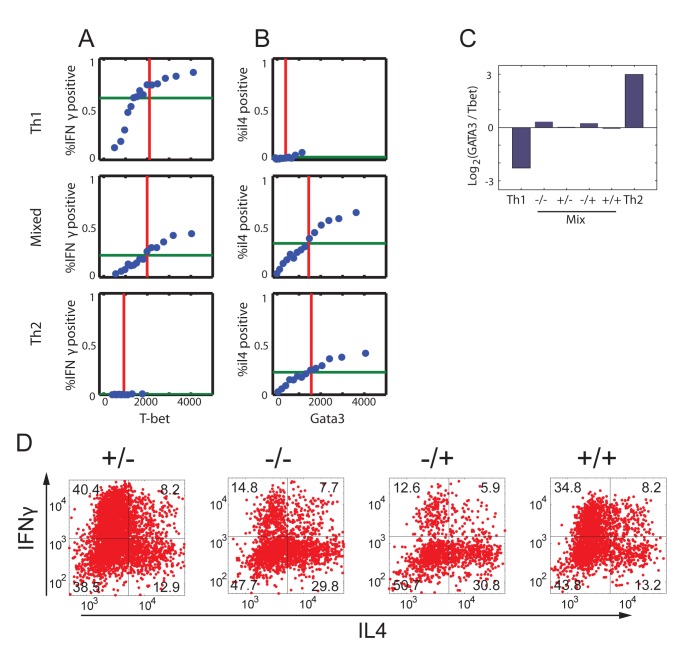
Heterogeneity in cytokine expression is generated through independent and biased stochastic processes. (A, B) Probability of cytokine expression is biased by the level of the corresponding TF. Cells growing under Th1, Mixed, and Th2 conditions were binned according to their measured level of (A) T-bet or (B) GATA3 expression. For each bin, containing 500 cells, the fraction of cells expressing (A) IFN-γ or (B) IL-4 is plotted versus the mean TF level of cells in that bin. The red line shows the population average level of the TF, and the green line shows the fraction of cytokine positive cells in the entire cell population. (C) GATA3/T-bet ratio is plotted for the four subpopulations of cytokine expression in cells growing under mixed conditions, compared with this ratio measured in cells cultured under polarizing Th1 or Th2 conditions. All four subpopulations of cells that were cultured under mixed conditions show a similar T-bet/GATA3 ratio, irrespective of their cytokine secretion state (−/−, +/−, −/+, +/+: corresponding to the four quadrants of Figure 4E). This observation is insensitive to the threshold values used to define the subpopulations (Figure S9). (D) Cells were cultured under mixed conditions for 1 wk, and then viably sorted into four subpopulations according to their cytokine expression pattern, as indicated (subpopulations correspond to the four quadrants of Figure 4E). Each subpopulation was re-cultured under mixed conditions for another week, restimulated, and levels of cytokine expression were measured, as shown. Within that week all subpopulations were able to re-populate all four quadrants, such that all cytokine expression patterns reappear.

These observation support a model of stochastic expression of IFN-γ and IL-4, as was previously observed for IL-4 and other cytokines [38]–[41]. Hence, a population of cells cultured under the same conditions is heterogeneous, with some cells expressing a cytokine while others do not. We set to characterize properties of stochasticity of those two cytokines, with respect to levels of expression of the two master regulators. As we measure the levels of the two TFs and two cytokines for each cell, our data allow for characterization of mutual dependencies between these proteins. Thus, we binned cells cultured under Th1, mixed, or Th2 conditions according to their level of T-bet or GATA3, and evaluated the chance of IFN-γ or IL-4 expression, respectively, in each bin. We find that the probability of cytokine expression monotonically grows with expression level of the corresponding TF (Figure 5A–B). Of note, although the probability of making IFN-γ in the entire population of cells grown under Th1-inducing conditions is ∼60% (Figure 5A top, green line), it reaches ∼85% for those cells expressing the highest levels of T-bet. Similarly, the probability of making IL-4 is ∼20% in the entire population of cells grown under Th2 conditions, while it reaches ∼40% in cells that highly express GATA3 (Figure 5B, bottom). Under mixed input conditions, both cytokines show a gradual monotonic increase in their probability of expression with the levels of their corresponding TF (Figure 5A,B, middle). Note that for Th1 (Th2)-inducing conditions, the GATA3 (T-bet) signal is mainly due to background (predominantly cell autofluorescence and nonspecific staining) and is uncorrelated with the secretion probability of the downstream cytokine, as expected. The results of Figure 5A,B suggest that stochastic expression of IFN-γ and IL-4 is biased by the level of expression of T-bet and GATA3, respectively.

Next, we checked for dependence between expression of the two cytokines in the same cell; for example: if a cell is expressing IFN-γ, does it have a higher or lower chance to express also IL-4? We find that expression of IFN-γ and IL-4 occurs by two independent stochastic processes (median mutual information MI = 0.023 over all input conditions, see also Figures S7 and S8). Similar results were obtained also for independence of IFN-γ and IL-13 (median MI = 0.005). These results are in accordance with previous studies that evaluated mRNA expression in T cell clones and in individual cells [42]. The Th2 cytokines IL-4 and IL-13 show somewhat larger dependence, though still at a low level (median MI = 0.04).

It was previously shown that IL-4-expressing and nonexpressing Th2 cells have similar levels of GATA3 [40]. We extend this analysis to include both the Th1 and Th2 axes, comparing levels of T-bet and GATA3 in the four subpopulations of cytokine expression. While absolute levels of the TFs somewhat vary between these subpopulations, we find that they all have a similar GATA3/T-bet ratio (Figures 5C and S9). Moreover, we observe that different cells that express the same levels of T-bet and GATA3 may show all four patterns of cytokine expression. Hence, although when cultured under mixed conditions some cells behave for example like Th1 cells (expressing IFN-γ but not IL-4) and others like Th2 cells (expressing IL-4 but not IFN-γ), their internal state, as defined by levels of expression of the master regulators, is mixed and similar. The distinction between cell state under mixed versus polarizing conditions is evident when comparing, for example, the subpopulation of cells that express IFN-γ but not IL-4. If taken from a population of cells that were cultured under Th1 conditions, the IFN-γ^+^–IL-4^−^ cells have high levels of T-bet and low levels of GATA3 (normalized GATA3/T-bet ratio ≪1, Figure S16). However, if taken from a population of cells cultured under mixed input conditions, both factors are expressed at high levels (normalized GATA3/T-bet ratio ∼1, Figure S16).

To further check stochasticity of cytokine production and stability of the mixed state, we viably sorted cells that were cultured under mixed conditions into four subpopulations, according to their expression pattern of IFN-γ and IL-4: −/−, +/−, −/+, and +/+. Each sorted subpopulation of cells was cultured for another week under mixed input conditions including T cell receptor stimulation. We find that all initial subpopulations are able to repopulate all four combinations of cytokine secretion following restimulation after the second week of culture (Figure 5D). In addition, all four sorted subpopulations retained their similar GATA3/T-bet ratio also after the second week of growth under mixed conditions, at an intermediate level (∼1), between those obtained for cells grown under Th1 and Th2 conditions (∼0.4 and ∼20, respectively, Figure S16B). We find some differences in the patterns of cytokine expression after the second week, between subpopulations of cells that expressed IFN-γ after the first week (+/−, +/+) and those that did not express it (−/−,−/+). The first show a higher tendency toward IFN-γ expression following the second week (Figure 5D). This difference may be attributed to the influence of the higher amounts of IFN-γ available for these cultures in the beginning of the second week, as it is expressed by the cells. This is different than the situation in the first week, where the cells only express lower amounts of IFN-γ upon primary activation. The excess amount of IFN-γ can drive cells stronger toward a Th1 phenotype, resulting in a higher fraction of IFN-γ-expressing cells and a lower fraction of IL-4-expressing cells. Nevertheless, the ability of cells sorted from the four subpopulations to repopulate all four states and the stability of the T-bet/GATA3 ratio provide evidence for further stability of the mixed state, for at least 2 wk of culture, though we cannot exclude convergence into the polarized states at longer times.

## Discussion

**Figure 6:**
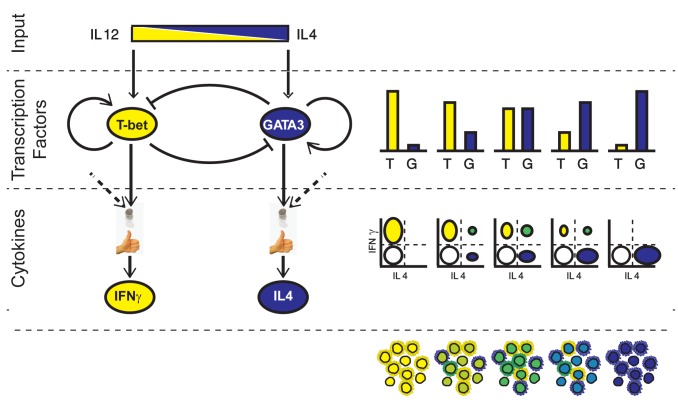
A two-stage scheme for continuously tunable Th1–Th2 differentiation. The two input signals (top) drive the GRN that controls differentiation of CD4^+^ T cells. The levels of the two lineage-specifying transcription factors, T-bet and GATA3, tune (bar graphs) from a Th1 state (left) to a Th2 state (right), through a continuum of intermediate states in which both factors are co-expressed. Cytokine expression upon restimulation is stochastic. The fraction of cells that express IFN-γ or IL-4 is biased by the levels of the corresponding transcription factors, as well as by other factors (dashed arrows). These two stochastic processes are independent. This model results in a heterogonous cell population (scatter plots, right), with cells expressing only IFN-γ (yellow ellipse), only IL-4 (blue), both cytokines (green), or neither (white). The fraction of cells in each of the four subpopulations continuously tunes with changing inputs. Expression levels of all four factors are represented schematically by the cell populations at the bottom. The internal color represents levels of T-bet and GATA3 tuning from Th1 (yellow, T-bet high, GATA3 low) to Th2 (blue, T-bet low, GATA3 high), through intermediate levels of green. The outer color represents cytokine expression upon restimulation, showing a higher level of heterogeneity. For clarity, we don't show here noise in gene expression (for example, cells cultured under Th1 conditions express different levels of T-bet, and similarly for the other proteins and conditions). Note that other factors influence this differentiation process (TCR stimulation strength and duration, other cytokines), which we assume here to be constant across all conditions.

Our findings can be explained by a two-stage model based on continuous, analogue expression of TFs that then bias a binary stochastic cytokine secretion. This model is shown schematically in Figure 6. First, input signals are mapped through the GRN in an analogue way, into continuously variable expression levels of the two master regulators. We show that the observed pattern of TF co-expression and their continuous tuning in response to levels of the input signals can be explained using the accepted simplified model for the network motif controlling the system, provided that the effective autoregulation on TF levels is graded, and dominates cross-inhibition. The second stage of the process is probabilistic in nature. During restimulation, cytokines are expressed stochastically in each cell with probabilities that are biased by the level of the relevant master regulator in that cell. These two stochastic processes are independent, as if the cell is throwing two biased coins, one determining whether to express IFN-γ or not, and the other determining whether to express IL-4 or not (see Figure S7). As each cytokine can be either “on” or “off,” under mixed conditions four subpopulations of cells arise, each with a different expression pattern: IFN-γ^−^/IL-4^−^, IFN-γ^+^/IL-4^−^, IFN-γ^−^/IL-4^+^, and IFN-γ^+^/IL-4^+^.

The model presented in this work can account for previous observations that challenged the Th1–Th2 dichotomy [43]. It can also reconcile the recent observations of continuums of hematopoietic cell states [18],[19] with prevailing theoretical models of cell differentiation. While the model predicts that cross-inhibition does not necessarily lead to multistability, it shows that it still restricts the state of the system. Thus, cells can be found only in a subset of the large multidimensional space defined by combinatorial protein expression, as restricted by the GRN. Within this allowed region, cell state can continuously tune, in response to levels of input signals.

While simplified models as we use here do not capture the full complexity of the regulatory network controlling cell differentiation, their main value is in their ability to reveal general classes of behaviour of these systems. Our analysis identifies conditions under which the widely used network motif of Figure 3A does not produce a bi- or tri-stable switch, but a mono-stable system, whose steady state continuously tunes from one extreme phenotype to the other in response to varying input levels. Our model can be refined when more quantitative data about the regulatory links controlling Th1–Th2 differentiation become available, together with detailed dynamical data on system state over time. Such approach was used, for example, to reveal the interplay between TCR and cytokine signalling during Th1 differentiation [44].

The core regulatory network that we studied (Figure 3A) was used previously to model the Th1–Th2 system [5],[6],[15],[17] as well as other systems of binary cell fate decisions [9],[11]–[14],[33]. A notable example is the PU.1–GATA1 system that controls erythroid/myeloid differentiation. Motivated by experimental results, various models of that network were proposed, which can generate tri-stability [9],[11]–[14]. Two of the steady states correspond to the two differentiated cell states (expressing either PU.1 or GATA1) and the third state corresponds to the progenitor cell state, in which both factors are expressed at intermediate levels. The levels of the two TFs in the progenitor state are highly variable between cells, and it was shown that this variability in turn biases the differentiation potential of progenitor cells [45],[46]. It is interesting to compare our results with those studies. While our data are better explained by one steady state when compared to a bi-stable system (Figure S14), we cannot exclude tri-stability with our current data. This is mainly due to the relatively high level of noise in the measurements of the expression of T-bet and GATA3 in single cells by flow cytometry. While part of this noise is technical, as can be seen from the T-bet knockout data (Figure 2A), the observed noise also reflects real biological variability between cells due to stochastic gene expression. A study in which mRNA molecules of GATA3 in Th2 cells were counted by single-molecule RNA fluorescence in-situ hybridization (FISH) supports our findings of highly heterogeneous GATA3 levels in the cell population [47]. Moreover, no bistability was observed for GATA3 mRNA levels in that study, supporting our observations at the protein level.

Although we cannot formally exclude tri-stability of the Th1–Th2 system, several differences between our observations and those of the PU.1–GATA1 system support a mono-stable tuneable solution in the case of Th1–Th2 differentiation. First, the observed mixed state is distinct from the progenitor state of the system, the naïve CD4^+^ T cell. Naive T cells show very low levels of expression of both T-bet and GATA3 [48], while in the mixed state both factors are expressed at levels similar to their levels in the polarized states (Figure 2C,D, Figure S1A,B). In addition, the mixed state requires simultaneous presence of the two inputs in order to up-regulate the expression of both transcription factors, unlike a progenitor state that is independent of the differentiation driving signals. Second, we observe stability of the mixed state: under mixed conditions, we detect cells that are co-expressing T-bet and GATA3 already 3 d after activation of naïve cells, and the levels of the two proteins at that time correlate with their levels at day 7 (Figure S2). Under mixed conditions, cells can be kept in culture for at least 2 wk while keeping expression of both transcription factors, and do not seem to resolve towards a more Th1 or Th2 like phenotype (Figure S16B). Finally, when binned into four subpopulations based on patterns of expression of the cytokines IFN-γ and IL-4, all four subpopulations (−/−, +/−, −/+, +/+) show similar GATA3/T-bet ratios after 1 wk (Figure 5C) and 2 wk (Figure S16B) of culture under mixed conditions. If the mixed population that we observe was a combination of cells in three stable states of a tri-stable system, one would expect to see a lower GATA3/T-bet ratio for cells that express IFN-γ but not IL-4 (+/−), and a higher ratio for cells that express IL-4 but not IFN-γ (−/+). Based on these observations, we conclude that the tuneable mono-stable model better explains our observations for the Th1–Th2 system compared to a tri-stable case. These results suggest that different cellular systems may use a similar gene circuit topology but have different dynamic properties, depending on the quantitative parameters of the regulatory network.

We have demonstrated how a gene regulatory circuit controlling cell fate decision can be designed for plasticity and robustness, to handle complex mixtures of signals to which cells are exposed. The continuously tuneable mixed states identified here can allow for a higher flexibility of the immune response under complex conditions when various counteracting signals may simultaneously occur. For example, in a recent study [49] it was shown that after infection causing a Th1 or Th2 response, most T cells in a draining lymph node were exposed to activating amounts of IFN-γ or IL-4, respectively. If Th1 and Th2 responses were mutually exclusive, T cells would lose their ability to respond to an unrelated challenge of the opposite nature that occurs simultaneously within the same lymph node. However, the mechanism described here allows cells to be in a mixed state, representing the actual levels of both signals. In this way, the system keeps the two options viable, at least until further information becomes available for these cells. Thus, cells can assess the two inputs and make a decision that is not binary but is gradual or fuzzy [50].

We suggest that this model of continuum differentiation combined with biased stochasticity is advantageous for differentiating systems that encounter uncertainty, such as most functions of the immune system [39],[51]. The classic bi-stable switch model is more suitable for rigid developmental programs such as embryonic development. We expect that similar continuously tuneable mixed phenotypes exist also in other differentiation pathways of CD4^+^ T cells (such as Th17, or induced regulatory T cells [52],[53]) and in other cell types in the hematopoietic system. We note that direct differentiation of naive CD4+ T-cells into a mixed Th1–Th2 phenotype was also observed concurrently by two other groups, using different experimental approaches [54],[55].

## Materials and Methods

### Ethics Statement

All animal work was approved by the Weizmann Institute's Institutional Animal Care and Use Committee (IACUC) and was conducted according to relevant national and international guidelines.

### Mice

Female 5–8-wk-old C57BL/6 mice were obtained from Harlan Laboratories (Rehovot, Israel) and housed at the Weizmann Institute. All mice were kept in small cages and fed sterile food and acid water.

### Cell Culture

Naïve CD4+ T-Cells were purified from C57BL/6 splenocytes by magnetic beads separation (CD4+CD62L+ MACS, Milthenyi biotech). As a control, we also sorted CD4+CD62L cells by FACS (FACSARia, BD), which provides higher purity of this population, avoiding potential memory phenotype cells. Similar results were obtained by both cell selection methods. Cells were cultured in a complete RPMI 1640 medium. CD4+ T cells were stimulated using plate coated with anti CD3 (1 µg/ml) and anti-CD28 (3 µg/ml) monoclonal antibodies in the presence of various external levels of IL-4 (0 to 540 ng/ml) and IL-12 (0 to 540 ng/ml) or the corresponding antibody (10 µg/ml), as indicated. After 4 d cells were removed from stimulations and transferred to a new plate for an additional 3 d. On day 7 cells were restimulated by a plate-bound anti-CD3 (2 µg/ml) for 4 h before addition of Brefeldin A and Monensin for an additional 2 h. Refreshing the media before restimulation was shown to have no significant effect on the results (Figure S10). Levels of T-bet and GATA3 somewhat increase upon restimulation, but are well-correlated before and after re-stimulation (Figure S15).

### Viable Cell Sorting

Cells were cultured under mixed cytokines condition (100 ng/ml IL12, 4 ng/ml IL4) as described above. On day 7 cells were stained with Miltenyi's cytokine secretion assay for IL-4 secretion (PE) and IFN-γ secretion (FITC), according to the manufacturer's instructions. Stained cells were sorted into four subpopulations of secretors/nonsecretors, using BD FacsAria (BD Biosciences). Secondary culture of the sorted groups was carried out for an additional 7 d as described above, while being exposed to mixed cytokine environment (100 ng/ml IL-12, 4 ng/ml IL-4).

### Flow Cytometry

Cells were stained with Invitrogen live/dead fixable dead cell stain kit. Subsequently cells were fixed and permeabilized, and were stained with various antibodies for 1 h at 4°C. The antibodies used were: FITC anti-IL-4, PerCP–Cy5 anti-IFN-γ, PacificBlue anti-T-bet, PE anti-CD4 (Biolegend), and Alexa647 anti-GATA3 (eBioscience). FACS analysis was performed using BD LSRII (BD Biosciences, Mountain View, CA).

### ELISA on Beads

Quantitative evaluation of protein levels in supernatant was done using an extension of the ELISA assay, with spectrally distinguishable beads (Spherotech PAK) as the solid phase. Primary antibodies were covalently linked to the beads, with spectrally different beads linked to antibodies against a unique cytokine (IFN-γ and IL-4). The various coated beads were incubated simultaneously with the supernatant and secondary biotinylated antibodies for 2 h, washed, and stained with streptavidin-PE. The beads' fluorescence level was used to separate the different cytokines. A standard curve for each cytokine was generated and fitted using a four-parameter hill function and was used to quantify fluorescent results (Figure S5). Beads were analyzed using BD LSRII.

### Flow Microscopy

Cells were stained with PacificBlue anti-T-bet, Alexa647 anti-GATA3, and APC/CY7 anti-CD4. The cells were run on the Imagestream X, an imaging flow cytometer that acquires up to six channels of imagery including brightfield, darkfield, and four channels of fluorescent imagery using a CCD camera. Images were analyzed using the Imagestream Data Analysis and Exploration Software (IDEAS 4).

### Data Analysis

Flow cytometry data were analyzed using “EasyFlow,” a dedicated in-house tool-set written in MATLAB, allowing graphical analysis of flow cytometry data, including gating, compensation control, histogram fitting, and statistical analysis, while providing a natural interface with native MATLAB algorithms. Cells were gated on four parameters: lymphocytes were selected using an FSC-SSC gate and live CD4+ cells were selected based on live/dead and anti-CD4 staining. The resulting cells were analyzed for protein levels. For population analysis, median fluorescence levels were calculated for each growth condition. Histograms were drawn using a logicle rescaling in such a way as to keep linearity for low fluorescence values while approaching log scale for high values [1]. For single cell analysis of secretion probabilities (Figure 5A,B), cells were binned according to their measured level of the transcription factor, and the percentage of cytokine-producing cells was calculated for each bin.

Data collected from three different experiments performed on different days are qualitatively similar (see Figure S11).

The angular parameters (see Figures 2G and 3F) were calculated in the log space. We shifted each parameter such that its isotype median is set to be at the origin (1,1) and then rescaled values such that the 95th percentile is normalized to 10. The angle was then calculated using the equation α = atan(x/y).

### RNA Extraction and cDNA Synthesis

Total RNA was extracted using RNeasy mini kit (Qiagen Valencia, CA), from the four sorted cell subpopulations (IL-4+, IFN-γ+, IL-4+ IFN-γ+, and nonsecreting cells). RNA concentration was measured on Nanodrop. 1 µg of total RNA was converted into cDNA using superscript II (Invitrogen)

### Quantitative TaqMan Real Time PCR

The ABI PRISM 7900HT Sequence Detection System was used for qt-RT-PCR analysis. Custom designed primer and probe sets were validated by serially diluting cDNA isolated from cells expressing the target gene and verifying the slope. Taqman PCR Master Mix was purchased from Applied Biosystems (NJ). Amplification was carried out in a total volume of 25 µl for 40 cycles of 15 s at 95°C, 1 min at 60°C. Initial denaturation was performed for 10 min at 95°C. Target gene expression was normalized relative to expression of the Abelson (ABL) gene.

## Supporting Information

Figure S1 Co-expression of TF and cytokine mRNA under mixed conditions.
(PDF)

Figure S2 Correlations between variables that define Th1 and Th2 response at the single cell level.
(PDF)

Figure S3 Measured input functions describing the lineage-specific TFs and cytokines show separation of variables.
(PDF)

Figure S4 Constant noise level of TFs for different input signals supports gradual response functions.
(PDF)

Figure S5 Cytokine secretion pattern follows the intracellular staining.
(PDF)

Figure S6 Cytokine secretion pattern continuously changes as a function of input signals.
(PDF)

Figure S7 INF-γ and IL-4 secretion after restimulation are two independent random processes.
(PDF)

Figure S8 The distribution of one cytokine expression level is independent of the other cytokine expression state.
(PDF)

Figure S9 GATA3/T-bet ratio in subpopulations is insensitive to threshold level.
(PDF)

Figure S10 Cytokine secretion is independent of external signal during restimulation.
(PDF)

Figure S11 Repeatability of experiments.
(PDF)

Figure S12 Theoretical model for the GRN controlling binary cell fate decision shows four different regimes if *n* = 1.
(PDF)

Figure S13 A complex network of known interactions controlling Th1/Th2 differentiation can be reduced into a simple toy model for the transcription factors.
(PDF)

Figure S14 Bayesian information criteria show that TF distributions are unimodal.
(PDF)

Figure S15 The profile of TF levels is established before restimulation.
(PDF)

Figure S16 GATA3/T-bet ratio is constant between the subpopulations and stable over 2 wk of culture.
(PDF)

Figure S17 A mixed state is observed also for IL-13, similar to IL-4.
(PDF)

Figure S18 Continuous tuning of the levels of IFN-γ, IL-4, and IL-13.
(PDF)

Table S1 References for the links in the GRN controlling Th1–Th2 differentiation (Figure S13).
(PDF)

Text S1 A parsimonious model of the GRN module governing cell differentiation shows a wide range of continuously tunable mono-stable solutions when positive feedback is gradual and dominates cross-inhibition.
(PDF)

## Abbreviations

FACS: fluorescence activated cell sorting
GRN: gene regulatory network
IFN: interferon
IL: interleukin
MFI: median fluorescence intensity
MI: mutual information
TF: transcription factor
Th1: T helper type 1
Th2: T helper type 2
