# Supplementary figures and images for: Mapping Differentiation under Mixed Culture Conditions Reveals a Tunable Continuum of T Cell Fates
Source: PLoS Biol. 2013 Jul 30;11(7):e1001616. doi: 10.1371/journal.pbio.1001616 (PMC3728017; doi:10.1371/journal.pbio.1001616)

Figure S1

**A**

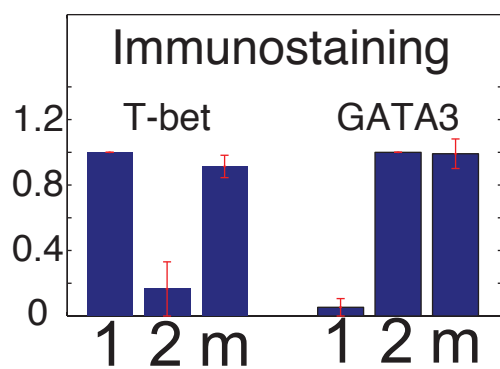

**C**

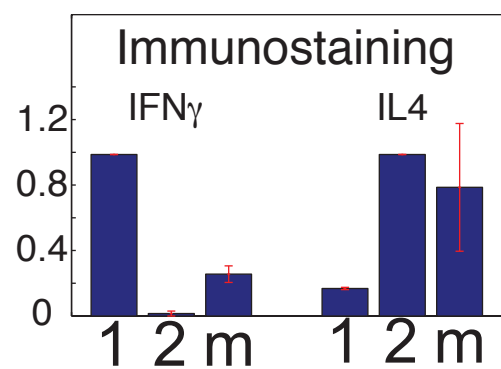

**B**

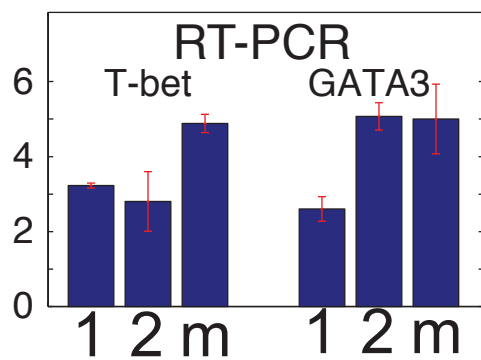

**D**

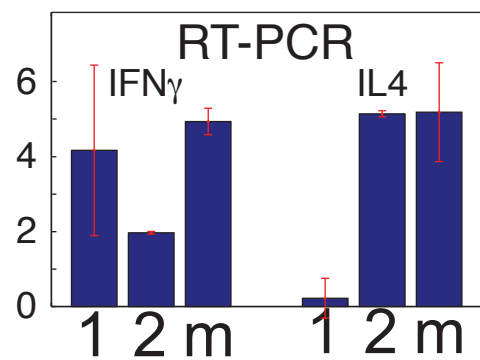

Supplement: Figure S1 — Co-expression of TF and cytokine mRNA under mixed conditions. (PDF) [file pbio.1001616.s001.pdf]

Figure S3

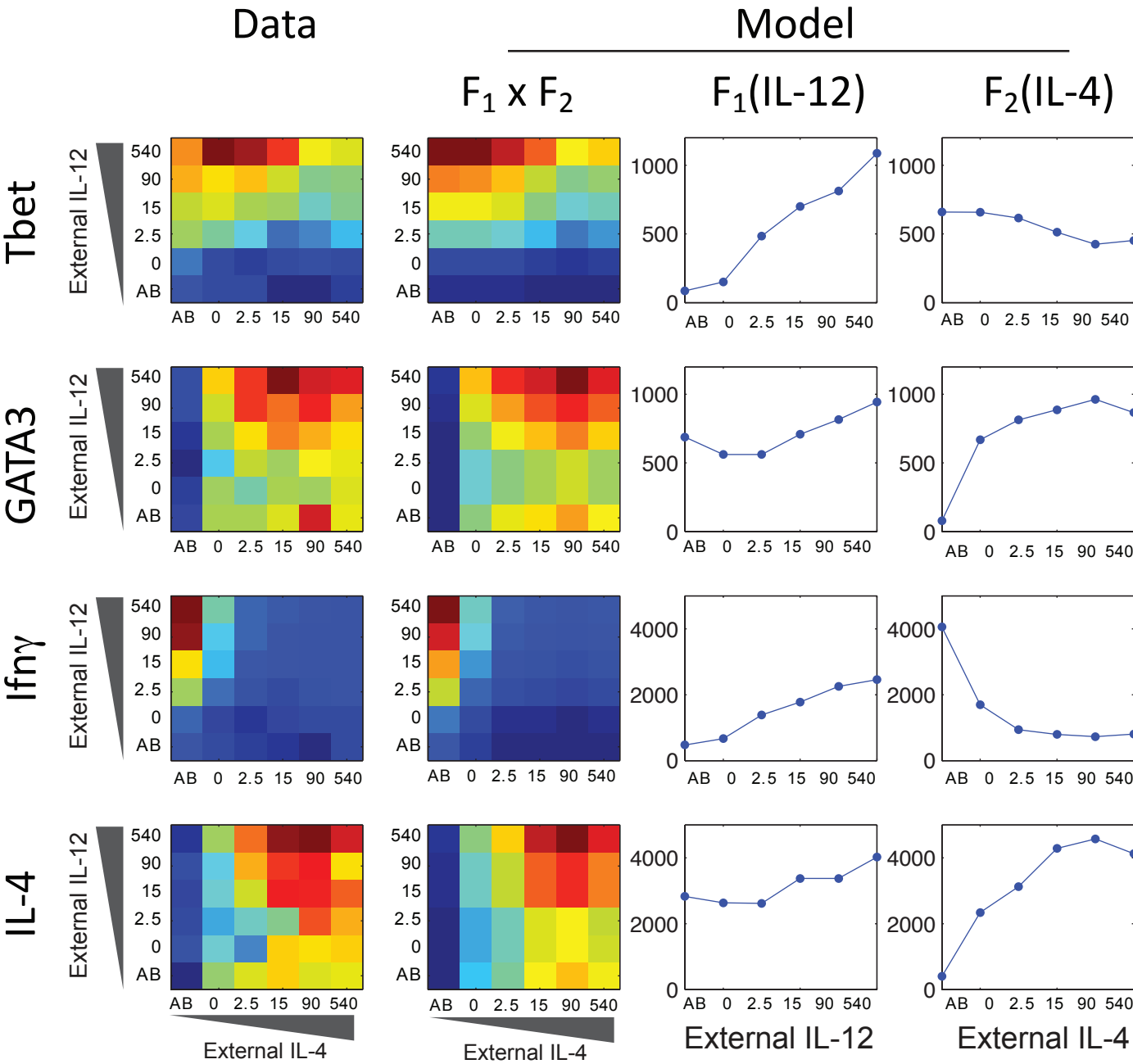

Supplement: Figure S3 — Measured input functions describing the lineage-specific TFs and cytokines show separation of variables. (PDF) [file pbio.1001616.s003.pdf]

Figure S4

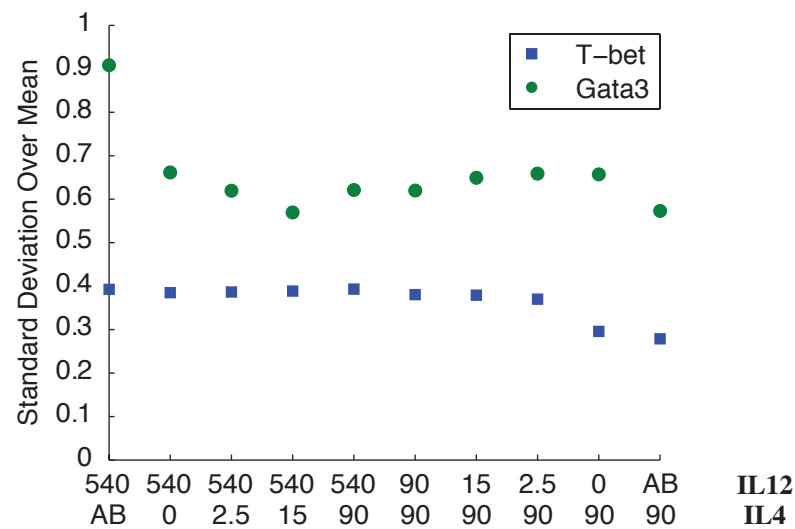

Supplement: Figure S4 — Constant noise level of TFs for different input signals supports gradual response functions. (PDF) [file pbio.1001616.s004.pdf]

Figure S5

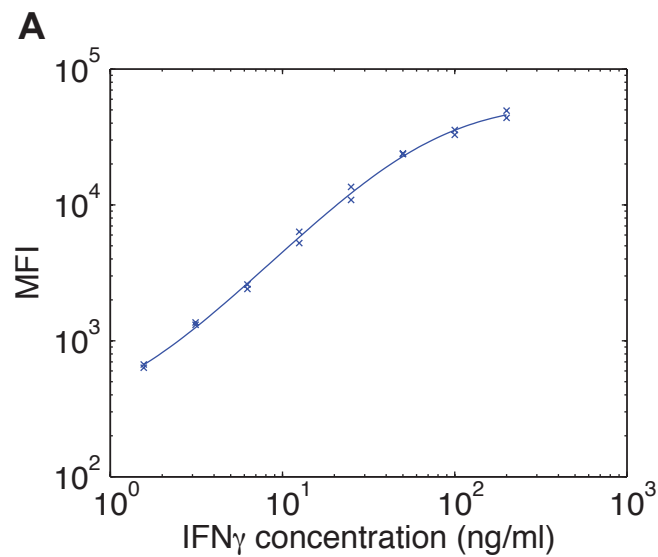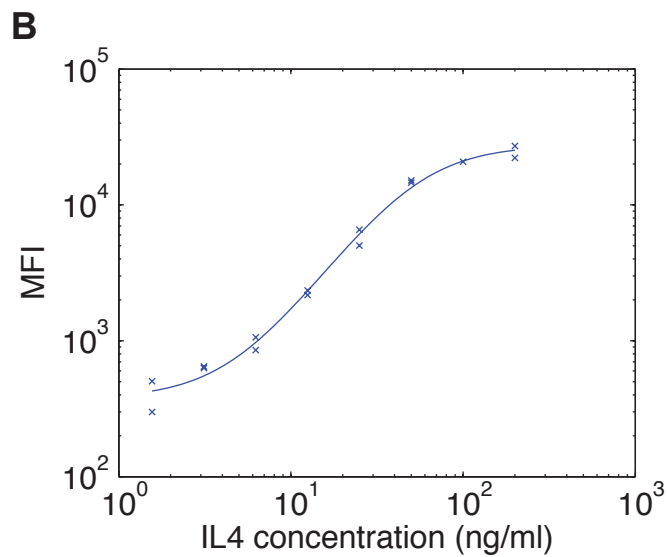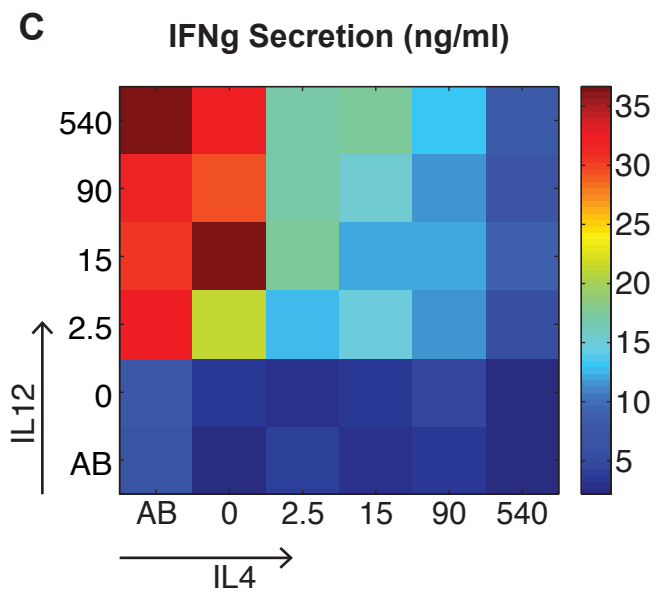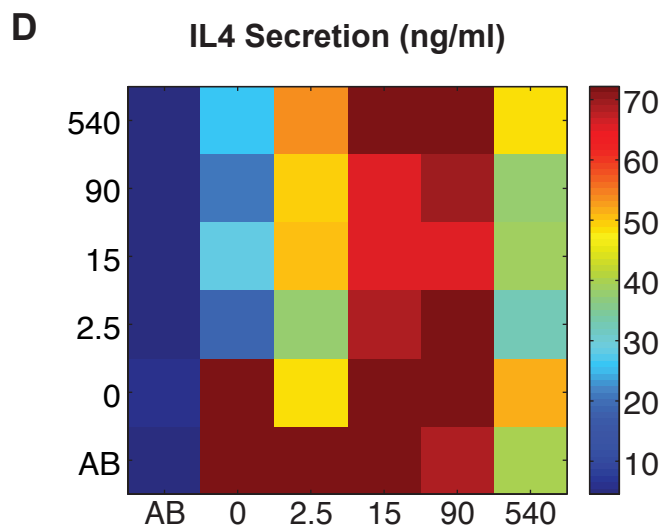

Supplement: Figure S5 — Cytokine secretion pattern follows the intracellular staining. (PDF) [file pbio.1001616.s005.pdf]

Figure S6

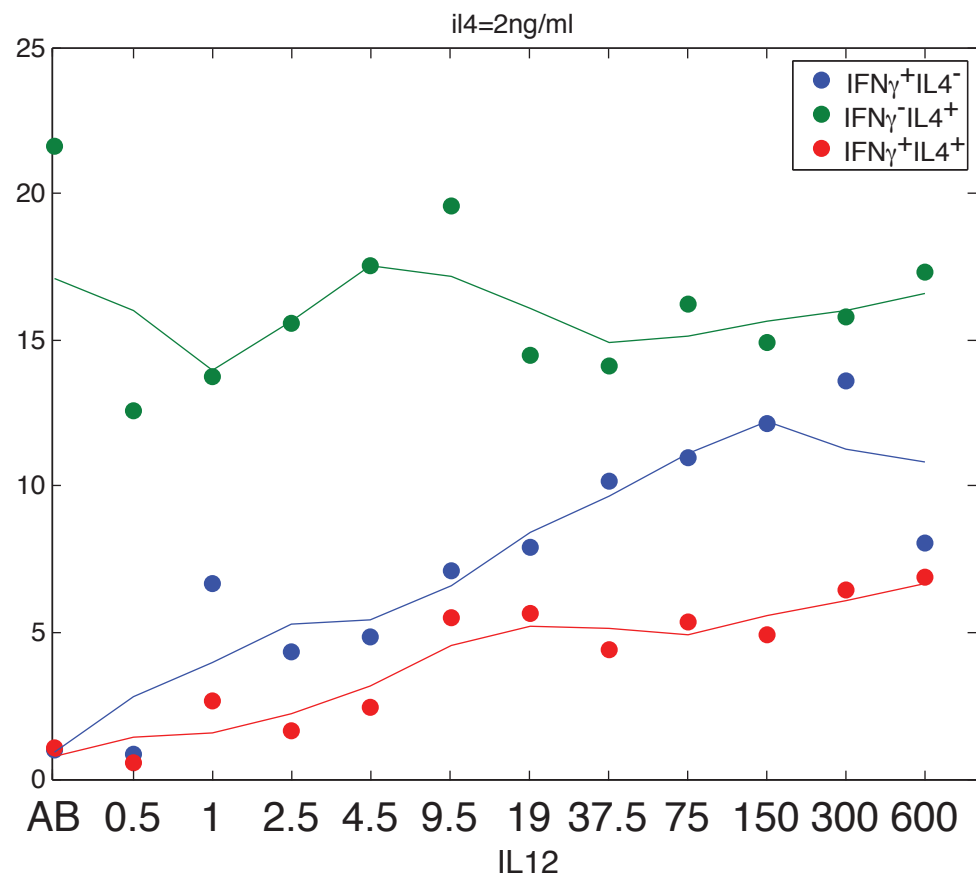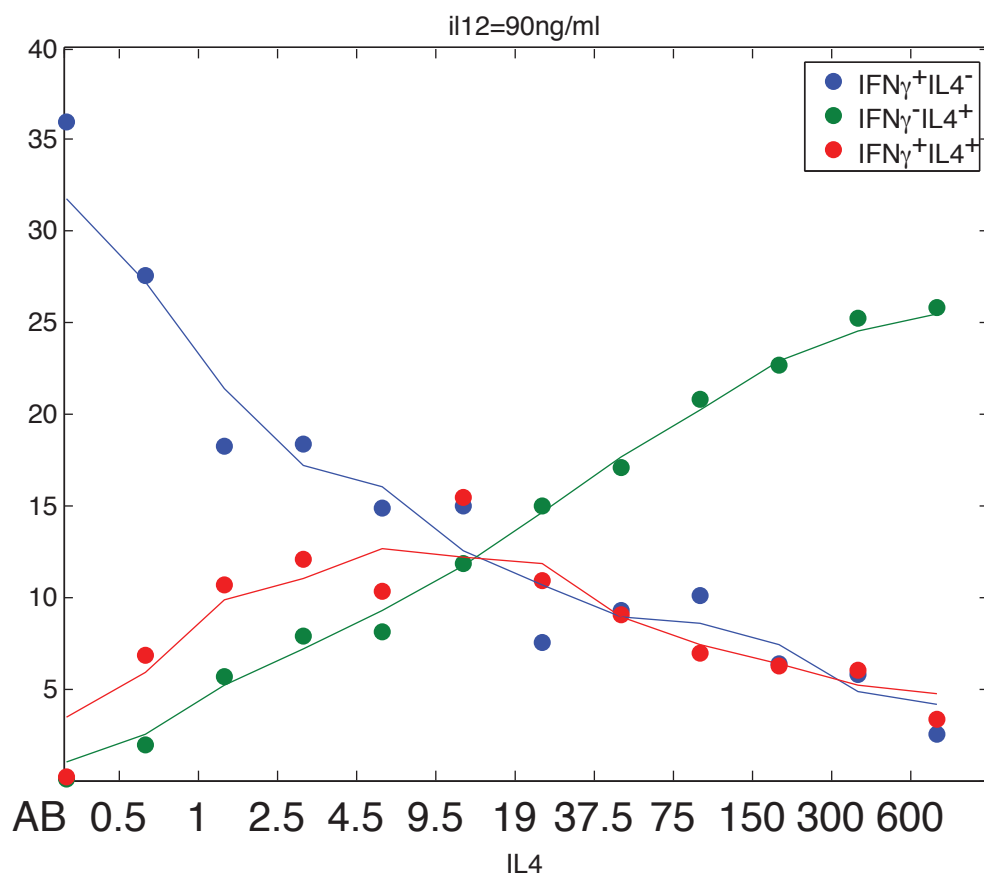

Supplement: Figure S6 — Cytokine secretion pattern continuously changes as a function of input signals. (PDF) [file pbio.1001616.s006.pdf]

**Figure S7**

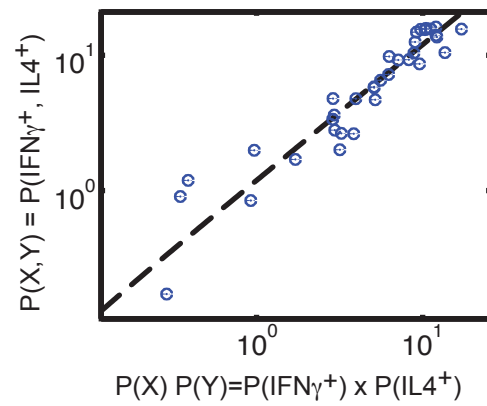

Supplement: Figure S7 — INF-γ and IL-4 secretion after restimulation are two independent random processes. (PDF) [file pbio.1001616.s007.pdf]

Figure S8

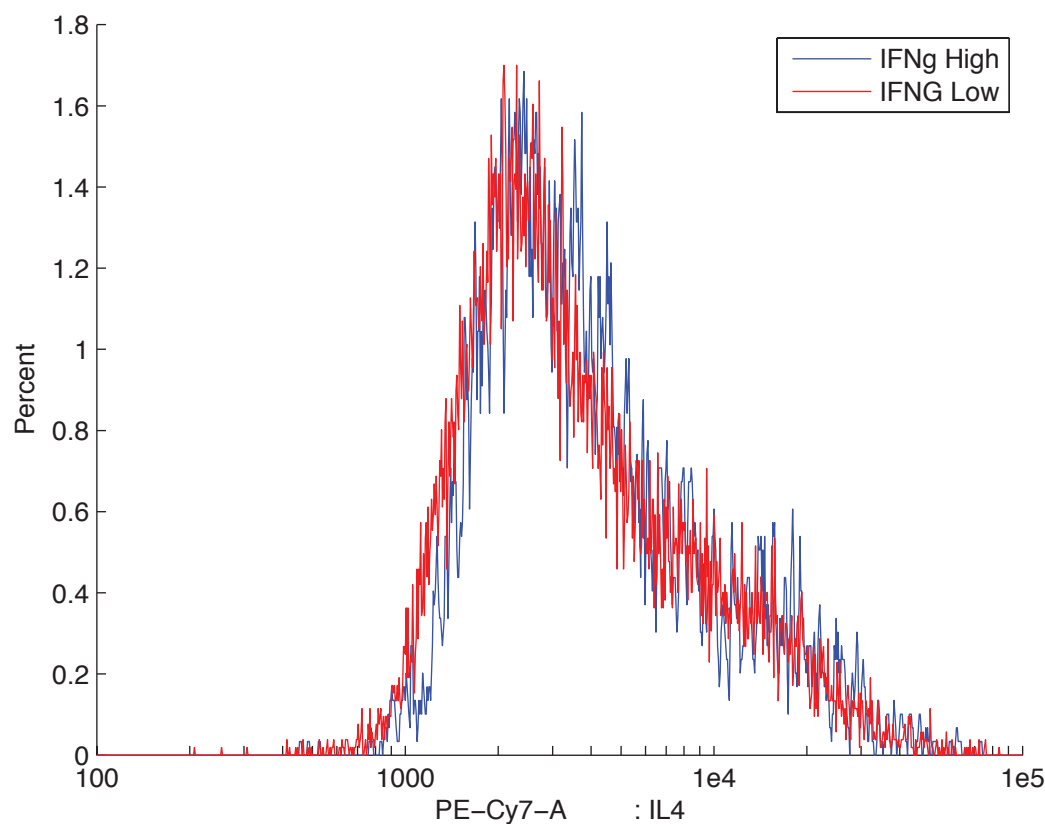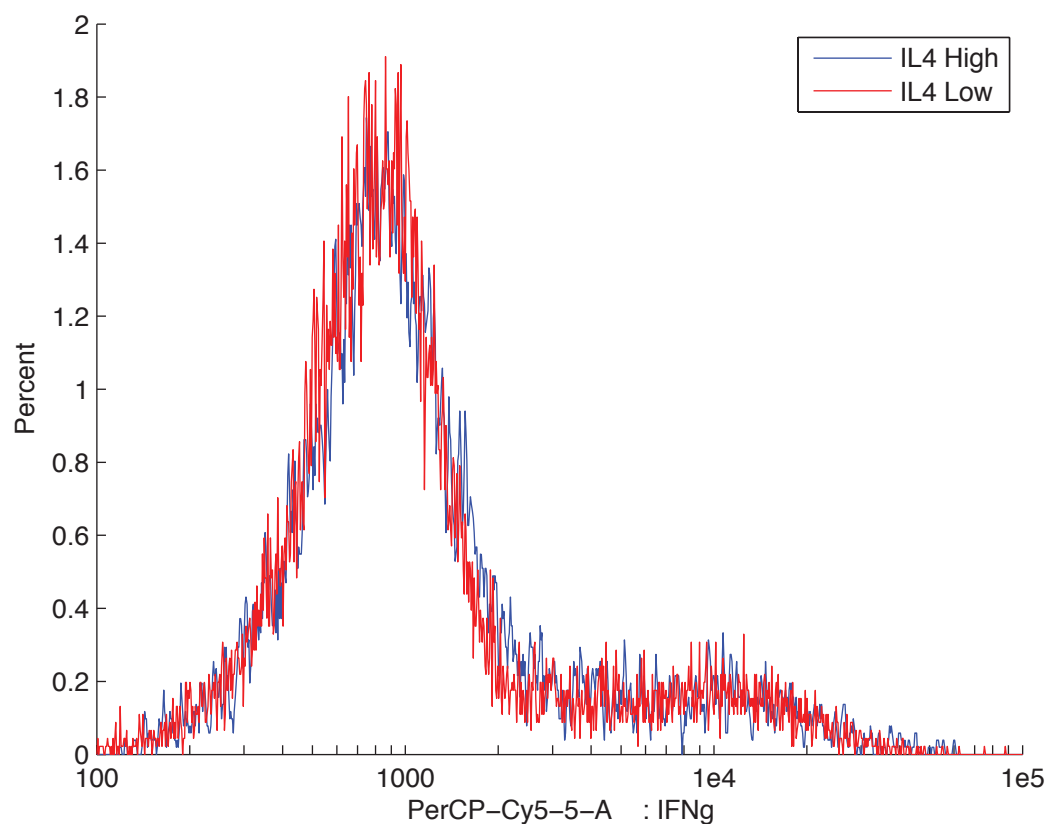

Supplement: Figure S8 — The distribution of one cytokine expression level is independent of the other cytokine expression state. (PDF) [file pbio.1001616.s008.pdf]

Figure S9

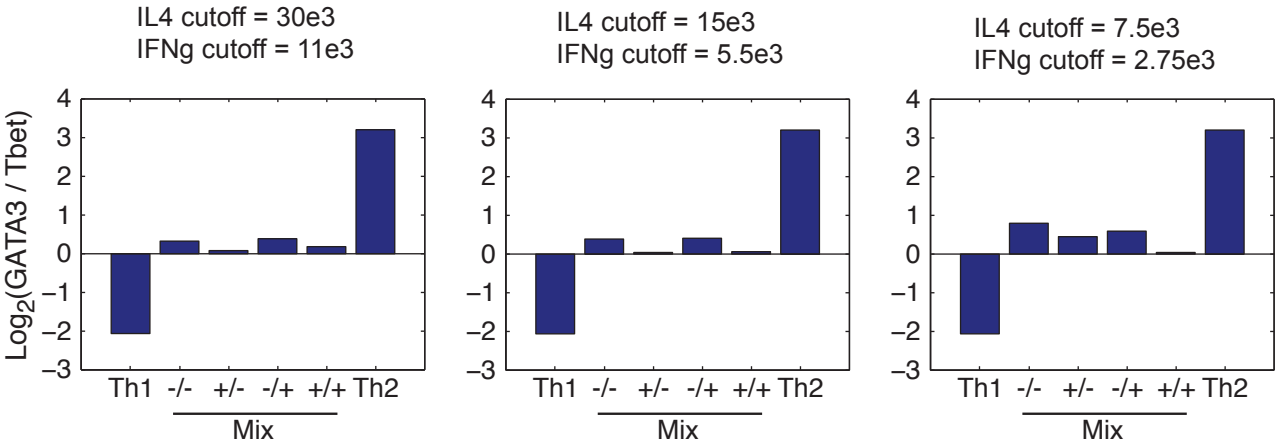

Supplement: Figure S9 — GATA3/T-bet ratio in subpopulations is insensitive to threshold level. (PDF) [file pbio.1001616.s009.pdf]

Figure S10

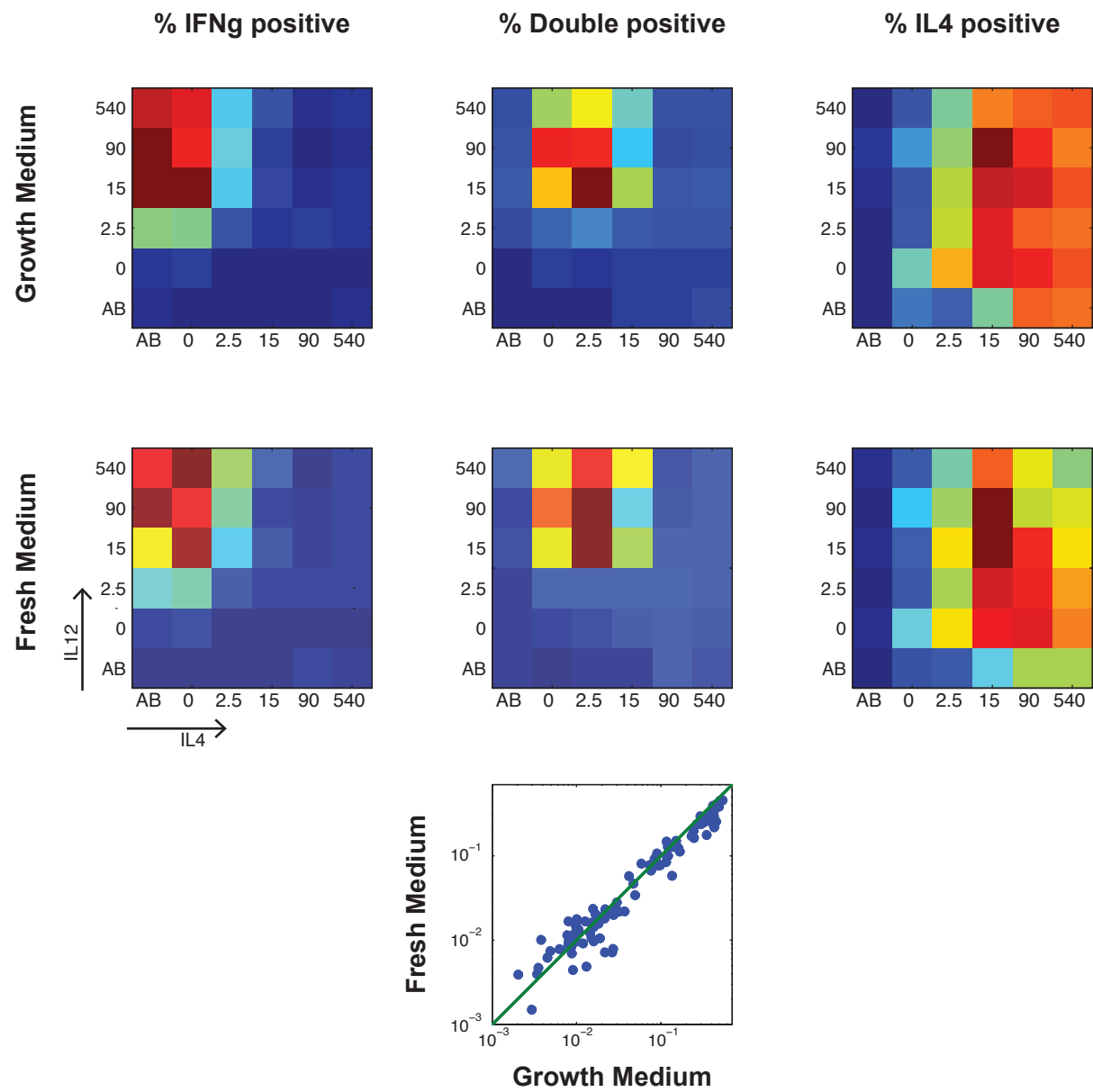

Supplement: Figure S10 — Cytokine secretion is independent of external signal during restimulation. (PDF) [file pbio.1001616.s010.pdf]

Figure S12

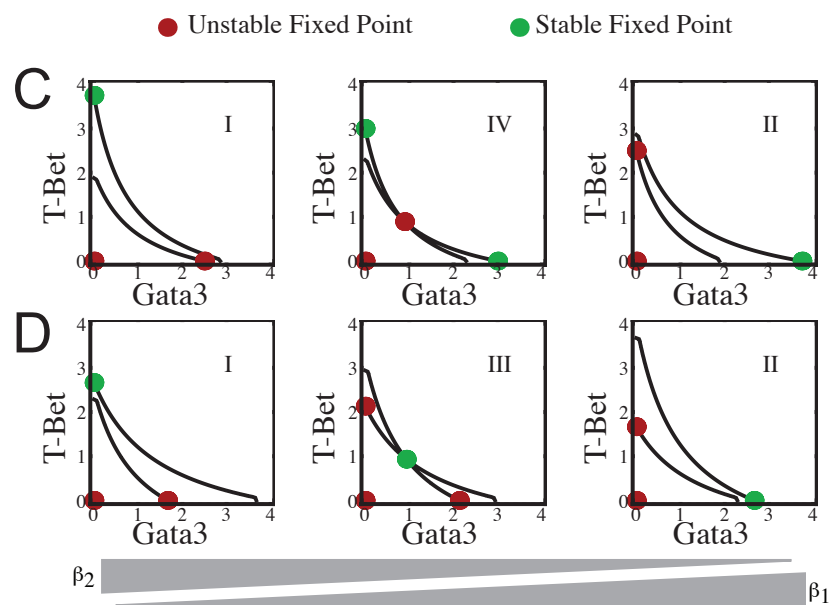

Supplement: Figure S12 — Theoretical model for the GRN controlling binary cell fate decision shows four different regimes if n = 1. (PDF) [file pbio.1001616.s012.pdf]

Figure S13

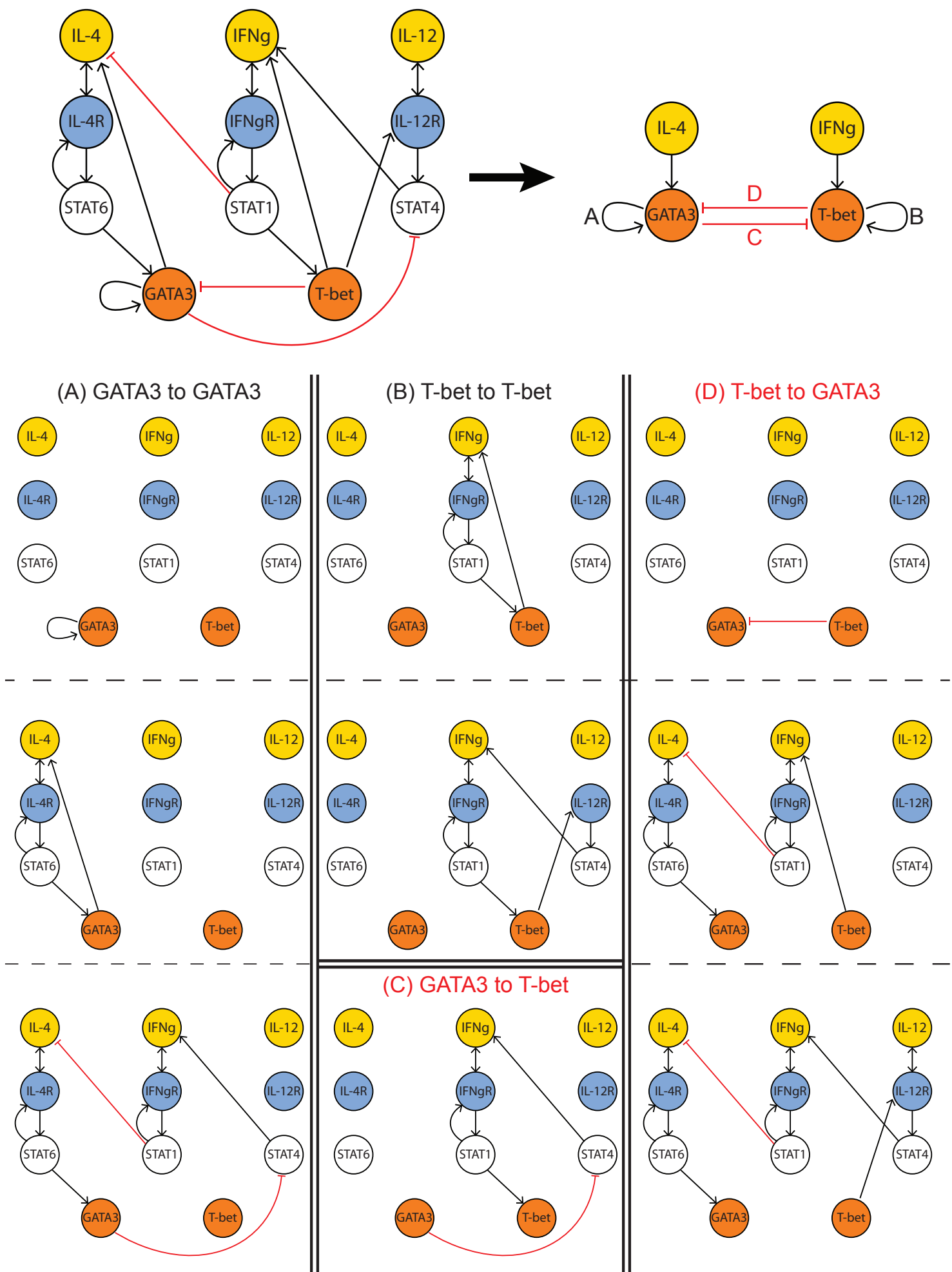

Supplement: Figure S13 — A complex network of known interactions controlling Th1/Th2 differentiation can be reduced into a simple toy model for the transcription factors. (PDF) [file pbio.1001616.s013.pdf]

Figure S14

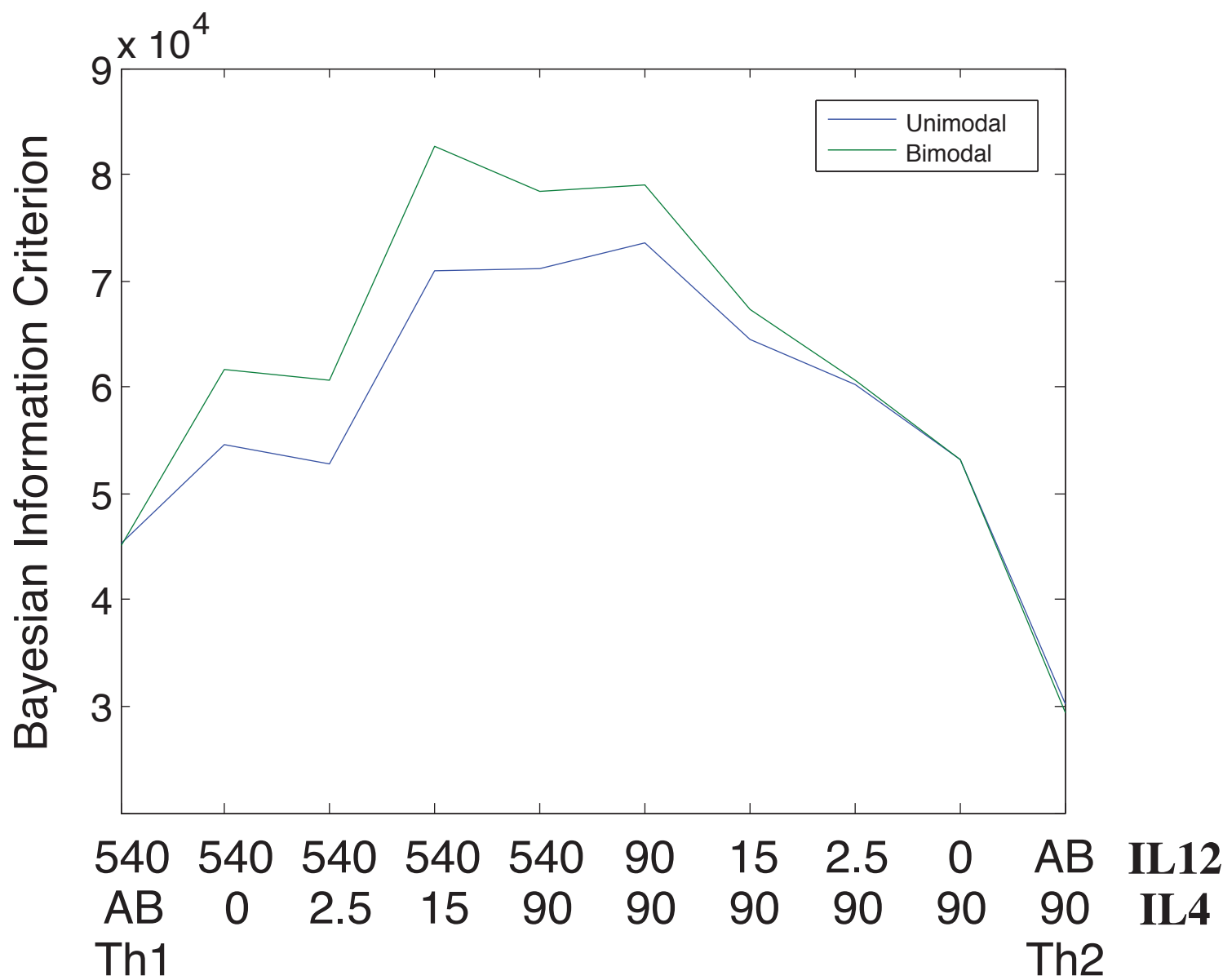

Supplement: Figure S14 — Bayesian information criteria show that TF distributions are unimodal. (PDF) [file pbio.1001616.s014.pdf]

Figure S15

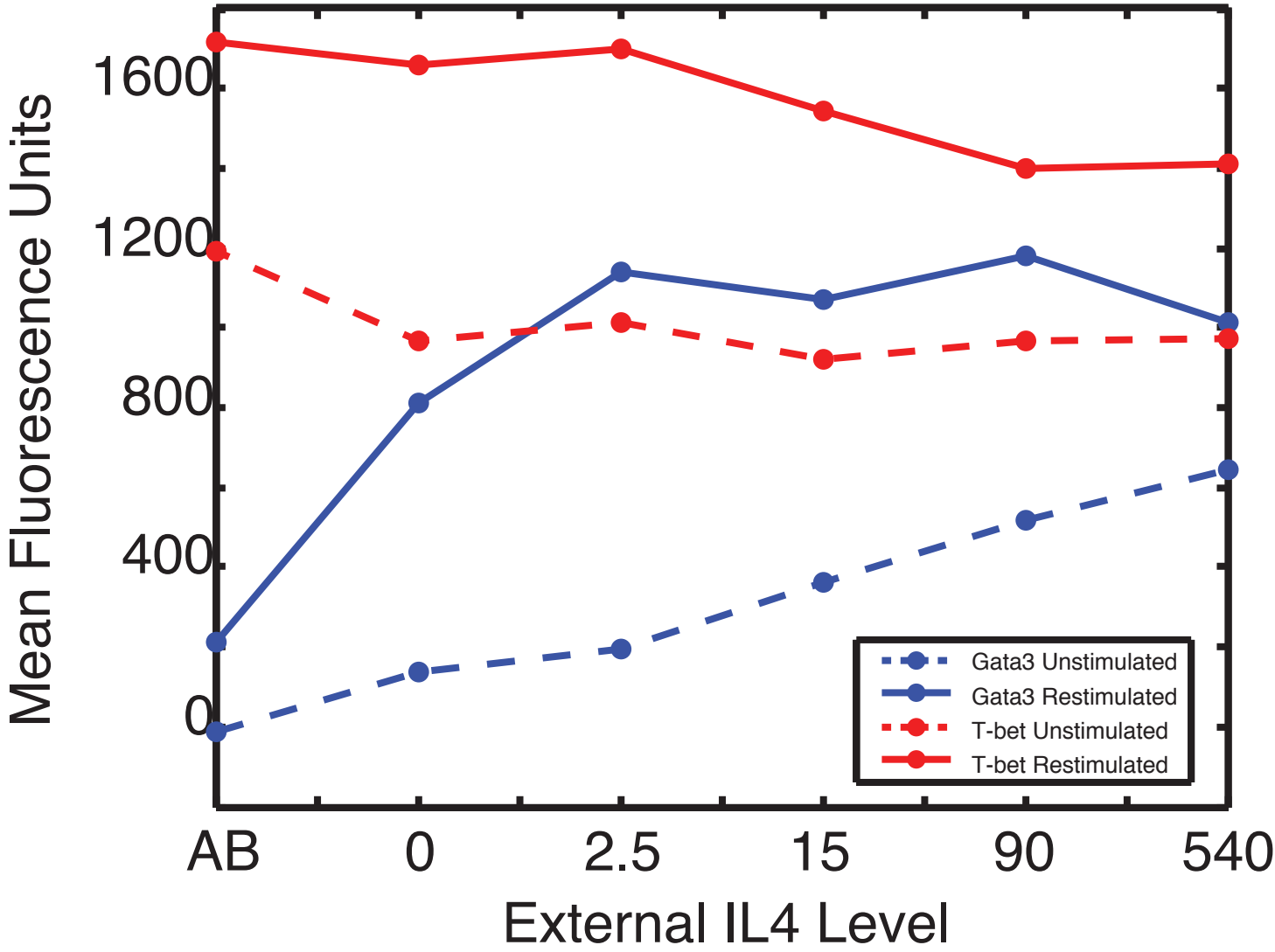

Supplement: Figure S15 — The profile of TF levels is established before restimulation. (PDF) [file pbio.1001616.s015.pdf]

Figure S16

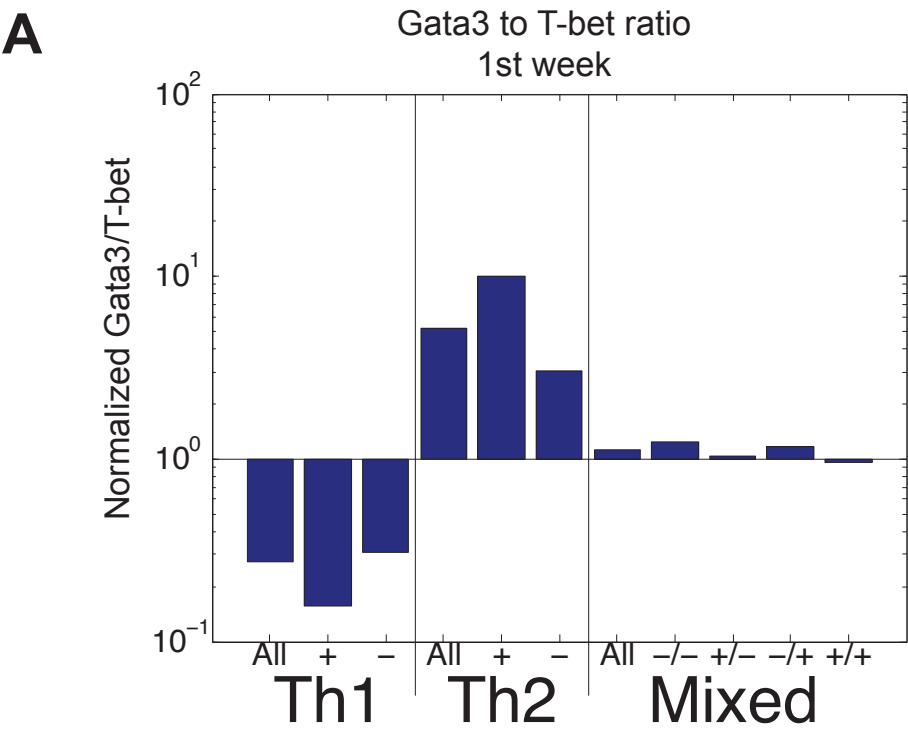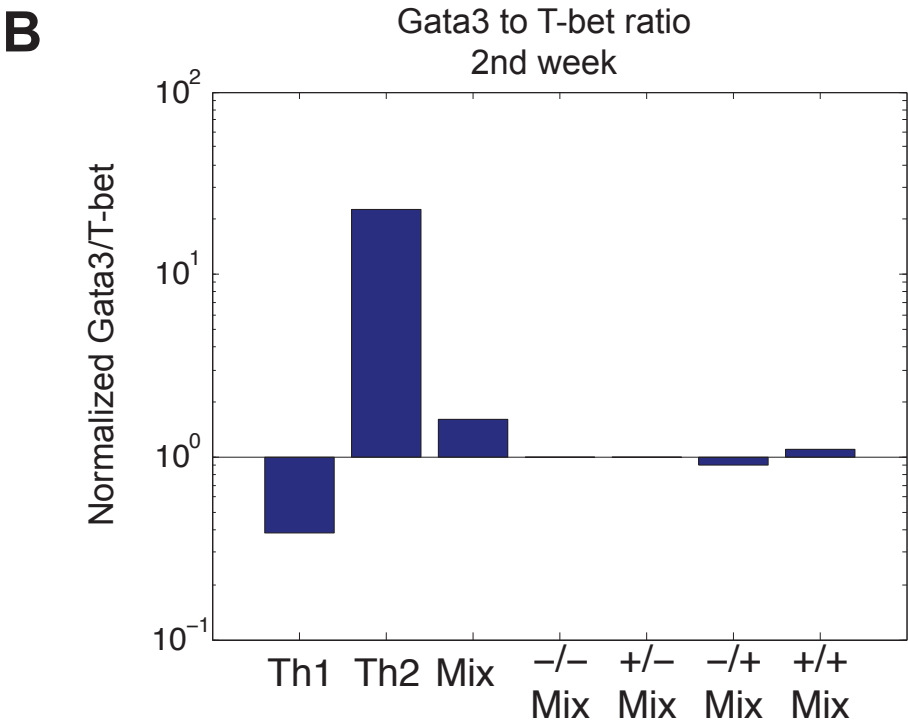

Supplement: Figure S16 — GATA3/T-bet ratio is constant between the subpopulations and stable over 2 wk of culture. (PDF) [file pbio.1001616.s016.pdf]

Figure S17

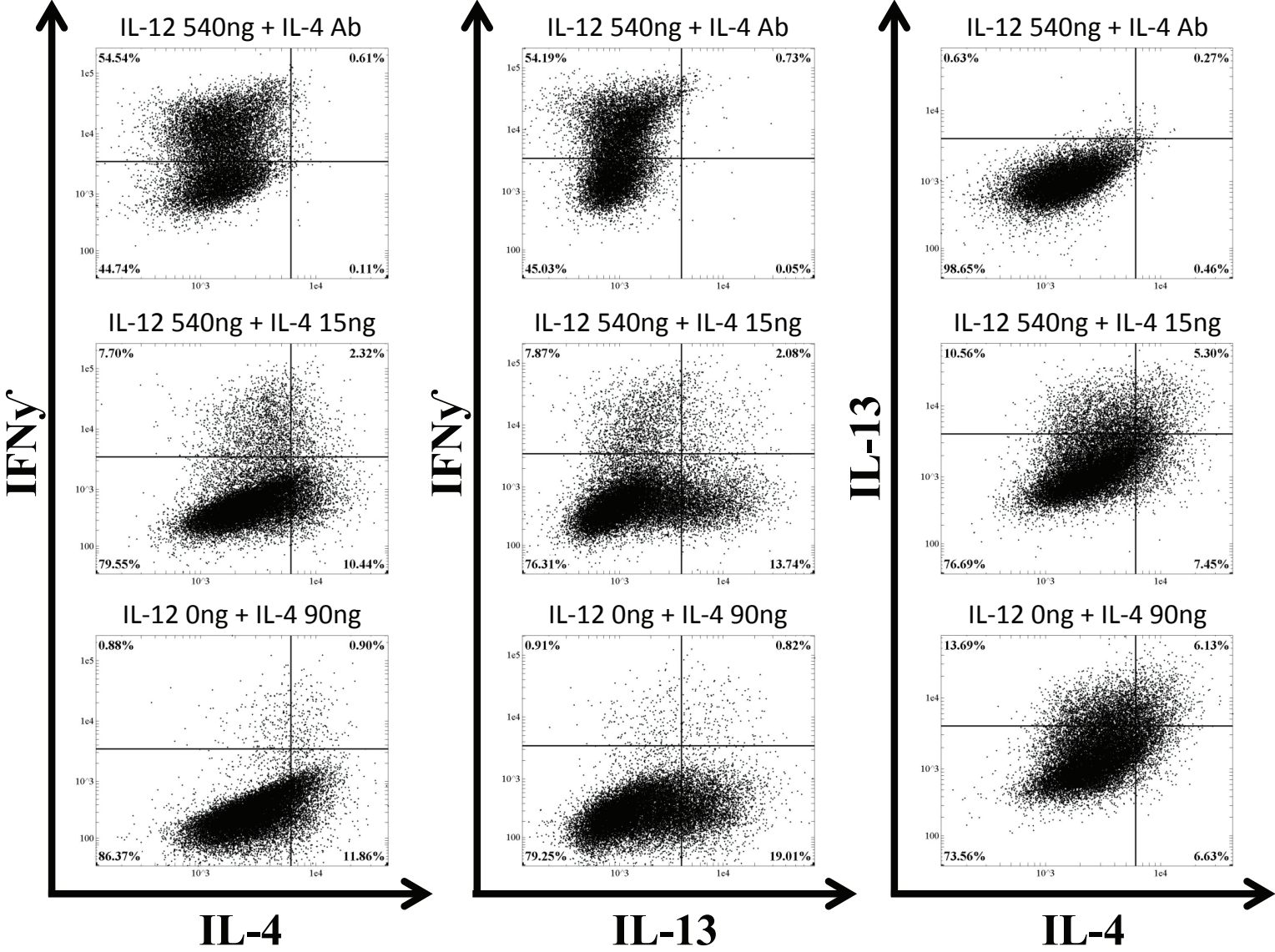

Supplement: Figure S17 — A mixed state is observed also for IL-13, similar to IL-4. (PDF) [file pbio.1001616.s017.pdf]

Figure S18

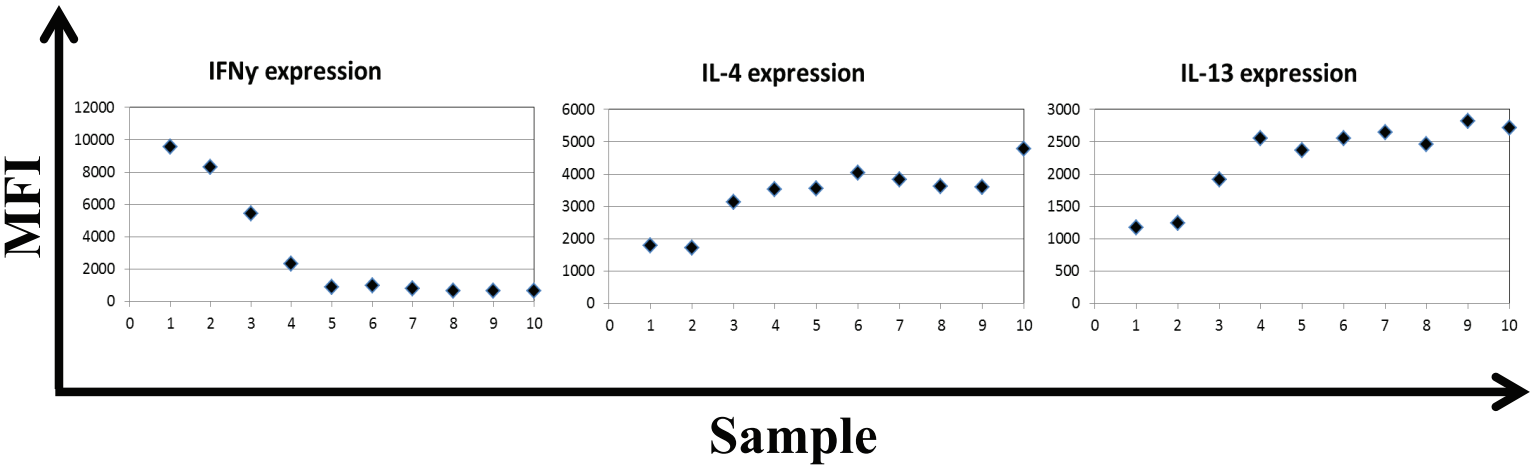

Supplement: Figure S18 — Continuous tuning of the levels of IFN-γ, IL-4, and IL-13. (PDF) [file pbio.1001616.s018.pdf]
