# Supplementary material for: Mapping Differentiation under Mixed Culture Conditions Reveals a Tunable Continuum of T Cell Fates
Source: PLoS Biol. 2013 Jul 30;11(7):e1001616. doi: 10.1371/journal.pbio.1001616 (PMC3728017; doi:10.1371/journal.pbio.1001616)
Supplement: Figure S2 — Correlations between variables that define Th1 and Th2 response at the single cell level. (PDF) [file pbio.1001616.s002.pdf]

Figure S2

Transcription Factors at day 3 and day 7

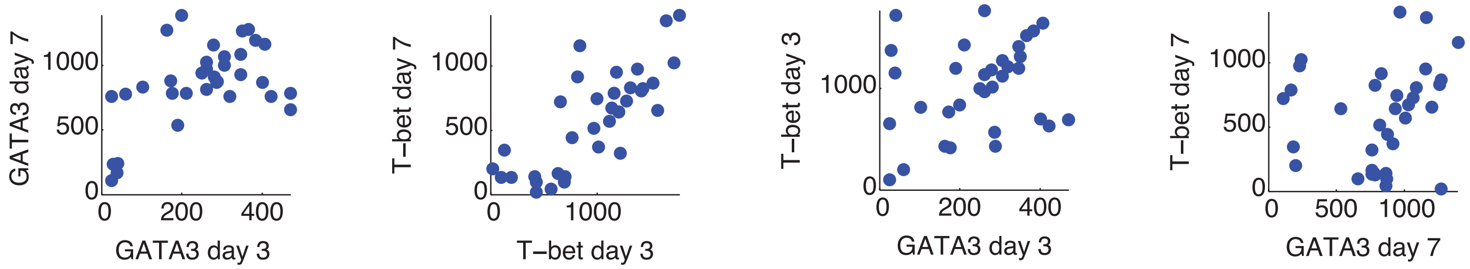

Cytokines vs. Transcription Factors

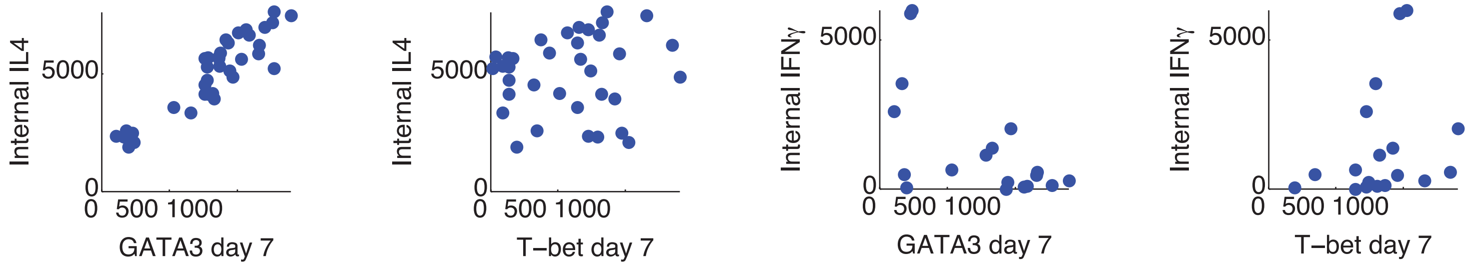

Internal vs. Secreted Cytokine Levels

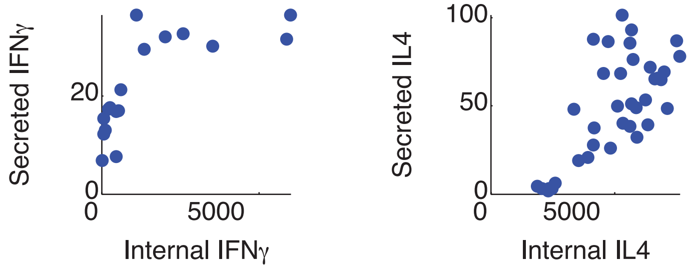

	GATA3 day 3	GATA3 day 7	Intra-cellular IL4	Secreted IL4	T-bet day 3	T-bet day 7	Intra-cellular IFNγ	Secreted IFNγ
GATA3 day 3	1	0.633	0.693	0.516	0.355	-0.04	-0.51	-0.37
GATA3 day 7	0.633	1	0.912	0.651	0.277	0.158	-0.57	-0.34
Intra-cellular IL4	0.693	0.912	1	0.705	0.267	0.092	-0.57	-0.41
Secreted IL4	0.516	0.651	0.705	1	-0.18	-0.37	-0.66	-0.61
T-bet day 3	0.355	0.277	0.267	-0.18	1	0.78	0.433	0.599
T-bet day 7	-0.04	0.158	0.092	-0.37	0.78	1	0.558	0.753
Intra-cellular IFNγ	-0.51	-0.57	-0.57	-0.66	0.433	0.558	1	0.811
Secreted IFNγ	-0.37	-0.34	-0.41	-0.61	0.599	0.753	0.811	1
