# Supplementary material for: Mapping Differentiation under Mixed Culture Conditions Reveals a Tunable Continuum of T Cell Fates
Source: PLoS Biol. 2013 Jul 30;11(7):e1001616. doi: 10.1371/journal.pbio.1001616 (PMC3728017; doi:10.1371/journal.pbio.1001616)

Figure S11

A

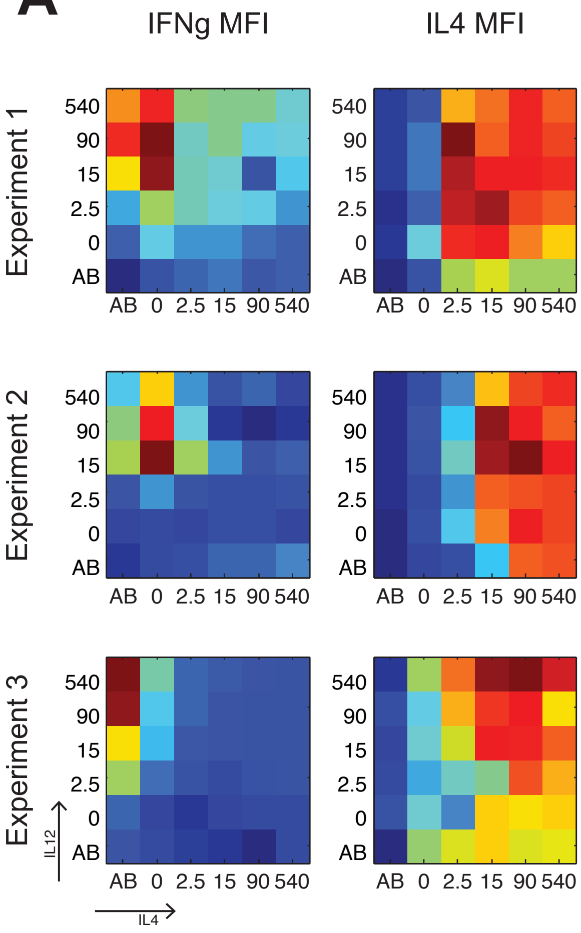

B

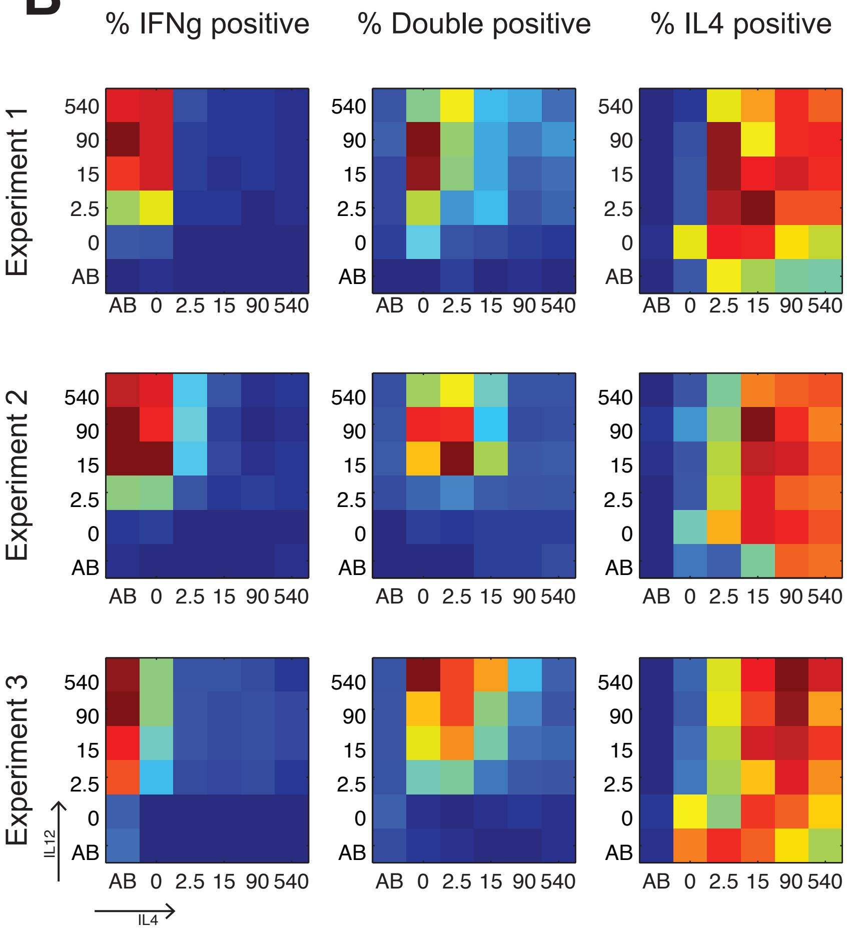

C

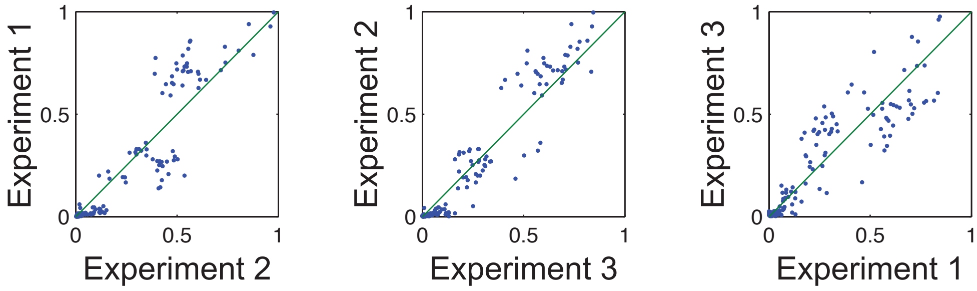

Supplement: Figure S11 — Repeatability of experiments. (PDF) [file pbio.1001616.s011.pdf]
