# Supplementary material for: Mapping Differentiation under Mixed Culture Conditions Reveals a Tunable Continuum of T Cell Fates
Source: PLoS Biol. 2013 Jul 30;11(7):e1001616. doi: 10.1371/journal.pbio.1001616 (PMC3728017; doi:10.1371/journal.pbio.1001616)
Supplement: Table S1 — References for the links in the GRN controlling Th1–Th2 differentiation (Figure S13). (PDF) [file pbio.1001616.s019.pdf]

**Table S1**

**References for the links in the gene regulatory network controlling Th1-Th2 differentiation (Fig. S13)**

| From | To | Source |
|---|---|---|
| IL-4R | STAT6 | M.H. Kaplan, U. Schindler, S.T. Smiley, and M.J. Grusby, Immunity, 4:313–319, 1996. |
| IL-12R | STAT4 | M.H. Kaplan, Y.L. Sun, , T. Hoey, and M.J. Grusby, Nature 382: 174–177, 1996. |
| IFN- γ R | STAT1 | C.V. Ramana, M. Chatterjee-Kishore, H. Nguyen, and G.R. Stark, Oncogene 19: 2619 - 2627, 2000. |
| STAT1 | Tbet | M. Afkarian, et. al., Nat. Immunol. 3: 549–557, 2002. |
| STAT1 | Secret IL-4 | B. Elser, et. al., Immunity 17:703–712, 2002. |
| STAT4 | Secret IFN-γ | W.E. Thierfelder WE, et. al., Nature, 382:171-4, 1996. |
| STAT6 | GATA3 | J. Zhu, H. Yamane, J. Cote-Sierra, L. Guo, W.E. Paul, Cell Research, 16: -10, 2006. |
| STAT6 | IL-4R | M.H. Kaplan, U. Schindler, S.T. Smiley, and M.J. Grusby, Immunity, 4:313–319, 1996. |
| GATA3 | Secret IL-4 | W.P. Zheng ,and R.A. Flavell, Cell 89:587 (1997). |
| GATA3 | STAT4 | T. Usui, R. Nishikomori, A. Kitani, and W. Strober, Immunity 18:415–28, 2003. |
| GATA3 | GATA3 | W. Ouyang, et al., Immunity 12:27–37, 2000. |
| Tbet | Secret IFN- γ | M. Afkarian, et. al., Nat. Immunol. 3: 549–557, 2002. |
| Tbet | GATA3 | E.S. Hwang et. al., Science 307:430-3, 2005. |
| Tbet | IL-12R | M. Afkarian, et. al., Nat. Immunol. 3: 549–557, 2002. |
