# Supplementary material for: Mapping Differentiation under Mixed Culture Conditions Reveals a Tunable Continuum of T Cell Fates
Source: PLoS Biol. 2013 Jul 30;11(7):e1001616. doi: 10.1371/journal.pbio.1001616 (PMC3728017; doi:10.1371/journal.pbio.1001616)
Supplement: Text S1 — A parsimonious model of the GRN module governing cell differentiation shows a wide range of continuously tunable mono-stable solutions when positive feedback is gradual and dominates cross-inhibition. (PDF) [file pbio.1001616.s020.pdf]

Text S1

A parsimonious model of the gene regulatory network module governing cell differentiation shows a wide range of continuously tunable mono-stable solutions when positive feedback is gradual and dominates cross-inhibition

In this section we describe the mathematical model we use for analyzing the gene regulatory network (GRN) regulating the transcription factors (TFs) involved in Th1-Th2 differentiation, T-bet and GATA3. The structure of the GRN is shown in Fig. 3A of the main text. In order to generate the model we have incorporated known interactions in the differentiation process of Th1-Th2 cells into a regulatory network diagram, similar to other published models of this system [2,3]. Similar models were used to study also other systems of binary cell fate decisions, and have shown that the steady states of the system exhibit bi- or tri-modality [2-7]. However, in contrast to previous studies, we identify a novel regime where a mono-stable solution exists. This holds when the positive autoregulatory loops for each of the TFs is gradual, giving rise to a wide dynamic range. In the model this translates to a low hill coefficient (of the order of 1) for each of the positive feedback autoregulatory loops.

The model assumes the following known interactions – T-bet (denoted as x) and GATA3 (denoted as y) each driving their own expression, creating a positive feedback loop while inhibiting the expression of the counterpart TF. These interactions are effective interactions and might represent an indirect sequence of interactions. The dynamic equations describing this model are given by:

$$(1)\ x' = x\left(\beta_1 \frac{x^{n-1}}{(K_x^n+x^n)(k_y^q+y^q)} - 1\right)$$

$$(2)\ \frac{1}{\gamma} y' = y\left(\beta_2 \frac{y^{m-1}}{(k_x^p+x^p)(K_y^m+y^m)} - 1\right)$$

Where K_x, K_y are the concentrations at which half activity is attained for the positive feedback of x and y respectively. Similarly, k_x, k_y are the concentrations at which half inhibition is attained for the negative cross-inhibition of x on y and y on x respectively. The factor γ is the ratio between the two degradation rates. We assumed a general hill function effect for all reactions of positive and negative kind.

The input signals driving the system are represented by the effective production rates of each of the TFs, β_1 and β_2. Experimentally these rates can vary by exposing cells to different levels of the external cytokines, IL-12 and IL-4, respectively. Without loss of generality, the rates were normalized in such a way that the degradation rate was set to equal one, note that this assumption does not change the structure of the phase space and can be relaxed.

We start by analyzing the case of no cooperativity, where all reactions are of the Michaelis-Menten form, m=n=p=q=1. Solving the model for the steady-state solutions one finds at most four fixed points:

(3) $x = 0, y = 0$

(4) $x = 0, y = \frac{\beta_1}{k_x} - K_y$

(5) $x = \frac{\beta_2}{k_y} - K_x, y = 0$

(6) $x = \hat{x}, y = \hat{y}$

where $\hat{x}, \hat{y}$ are the solutions of the quadratic equation

(7) $$\left(\beta_1 \frac{1}{(K_x+\hat{x})(k_y+\hat{y})} - 1\right) = \left(\beta_2 \frac{1}{(k_x+\hat{x})(K_y+\hat{y})} - 1\right) = 0.$$

The four solutions represents the OFF state (Eq. 3), where no TF is expressed, the two fully polarized solution (Eqs. 4-5) where only one TF is expressed while the other is inhibited, and the novel mixed state (Eq. 6) where both TF are expressed simultaneously. The stability of the different fixed points is set by the ratios $\tilde{k}_1 = \frac{k_y}{K_x}, \tilde{k}_2 = \frac{k_x}{K_y}$ (i.e. the relative strengths of negative vs. positive feedback for each TF) and depends also on the values of the two inputs, β_1 and β_2. Systematic analysis of the parameter phase space reveals several regimes with different stability patterns. In general we find four different such regimes (Fig. 3, Fig. S13). The two extreme regimes (I and II), where one signal is much stronger than the other give rise to a single stable fixed point describing either Th1 (for $\beta_1 \gg \beta_2$, regime I) or Th2 (for $\beta_2 \gg \beta_1$, regime II). In the intermediate regimes (III and IV), where the signals are similar, we find two different behaviors: bimodality or a monostable mixed state. In the first, both polarized states (Eqs. 4 and 5) are stable simultaneously (regime III), while in the second, the only fixed point is the mixed state (Eq. 6) (regime IV). As we vary β_1 and β_2, the point of separation between the bi-stable region and the mono-stable mixed state moves along a hyperbola on the $\tilde{k}_1 - \tilde{k}_2$ space. Its location is given by the following relation

(8) $\tilde{k}_1 = \frac{1}{\tilde{k}_2}, \tilde{k}_2 = \frac{1+\beta_2}{1+\beta_1}$.

For example, when $\beta_1 = \beta_2$ the point of separation between the two regions lies at $(\tilde{k}_1, \tilde{k}_2) = (1,1)$. This situation is shown in Fig. 3B.

The phase space can be probed by exposing cells to different external conditions, thereby changing β_1 and β_2. By gradually moving from Th1 supporting conditions (regime I) to Th2 supporting conditions (regime II) the system must go through one of the transition regimes in which it exhibits either bi-stability or a mixed-phenotype behavior. As varying the β's doesn't change the feedback parameters, the system either lies above the

hyperbola described in equation 8, or below it. As this is the separating point between the two transition regimes the expected behavior would depend on parameters of the feedback alone. Therefore by measuring the behavior for a set of initial conditions interpolating between Th1 conditions and Th2 conditions we will always see the same behavior for a given system. Varying input signals cannot change a bi-stable system into a mixed phenotype one and vice versa. Note that when $\beta_1 \sim \beta_2$, there is a region of parameters where equation 6 has two solutions. This gives rise to a new regime with bi-stability where one of the solutions is polarized while the other exhibits mixed TF expression. As this region is not extended and covers a relatively very small area of the phase space (being at most 10% of the above mentioned bi-stable region with a median of 1% for β_1 / β_2 ratio ranging between 1 to 5) it requires a fine tuning of the external signals in order to pass through this regime. This is in contrast to the previously mentioned regimes, which any path connecting the two polarizing conditions must cross.

Further numerical analysis shows that increasing the hill coefficients of the negative cross-inhibition links (q and p in Eq. 1 and 2, respectively) does not change these results significantly, but rather increases the phase space range where a mono-stable solution is attained. This can be understood intuitively since increasing the negative feedback shifts the inhibitory response towards a step function, thus yielding a larger regime where the mutual inhibitory influence is weak thus allowing the two TF concentrations to build up to a non-zero value. Thus, increasing the hill coefficient of the negative cross-inhibition links does not change qualitatively the phase-space of mono-stability vs. bi-stability.

Next, we examine the case where the positive autoregulatory feedback is greater than one. In this case, as shown previously by a number of studies [2-7], the dynamic behavior is mostly bi-stable by nature, as can be seen from analyzing the Jacobian of the system:

$$(9)\ J = \begin{pmatrix} -1 + \frac{n\,\beta_1\,x^{n-1}}{(1+x^n)^2(k_y+y^p)} & -\frac{p\,\beta_1\,x^n y^{p-1}}{(1+x^n)\left(k_y+y^p\right)^2} \\ \frac{q\,\beta_2 x^{q-1}\,y^m}{(k_x+x^q)^2(1+y^m)} & -1 + \frac{m\,\beta_2\,y^{m-1}}{(k_x+x^q)(1+y^m)^2} \end{pmatrix}$$

One can see that the point (0,0) is always a stable fixed point of the system. Furthermore, the two polarized fixed-points points $(x_p^*, 0)$ and $(0, y_p^*)$ can be stable together given that the parameters hold

$$(10)\qquad \frac{n\,\beta_1}{k_y}\frac{{x_p^*}^{n-1}}{\left(1+{x_p^*}^n\right)^2} < 1, \frac{m\,\beta_2}{k_x}\frac{y^{m-1}}{(1+y^m)^2} < 1$$

Therefore, in the case of higher hill coefficients for the positive feedback loop ($n, m \geq 2$), there is a wide range of parameters such that the two fully polarized fixed points are both stable, leading to a large regime of bi-stability (which stands in contrast to the case of the positive feedback loops having hill coefficients equal 1, as shown above).

Supplementary references

1. D.R. Parks, M. Roederer, W.A. Moore, "A new "Logicle" display method avoids deceptive effects of logarithmic scaling for low signals and compensated data", Cytometry A. 69,541-51 (2006).

2. L. Mariani, M. Löhning, A. Radbruch, T. Höfer, Transcriptional control networks of cell differentiation: insights from helper T lymphocytes., *Progress in biophysics and molecular biology* **86**, 45-76 (2004).

3. A. Yates, R. Callard, J. Stark, Combining cytokine signalling with T-bet and GATA-3 regulation in Th1 and Th2 differentiation: a model for cellular decision-making., *Journal of theoretical biology* **231**, 181-96 (2004).

4. S. Huang, Y.-P. Guo, G. May, T. Enver, Bifurcation dynamics in lineage-commitment in bipotent progenitor cells., *Developmental biology* **305**, 695-713 (2007).

5. I. Roeder, I. Glauche, Towards an understanding of lineage specification in hematopoietic stem cells: a mathematical model for the interaction of transcription factors GATA-1 and PU.1., *Journal of theoretical biology* **241**, 852-65 (2006).

6. M. Andrecut, J. D. Halley, D. A. Winkler, S. Huang, A general model for binary cell fate decision gene circuits with degeneracy: indeterminacy and switch behavior in the absence of cooperativity., *PloS one* **6**, e19358 (2011).

7. J. Wang, K. Zhang, L. Xu, E. Wang, Quantifying the Waddington landscape and biological paths for development and differentiation., *Proceedings of the National Academy of Sciences of the United States of America* **108**, 8257-62 (2011).
